# The genomic landscape of canine osteosarcoma cell lines reveals conserved structural complexity and pathway alterations

**DOI:** 10.1371/journal.pone.0274383

**Published:** 2022-09-13

**Authors:** Kate Megquier, Jason Turner-Maier, Kathleen Morrill, Xue Li, Jeremy Johnson, Elinor K. Karlsson, Cheryl A. London, Heather L. Gardner

**Affiliations:** 1 Broad Institute of MIT and Harvard, Cambridge, Massachusetts, United States of America; 2 University of Massachusetts Chan Medical School, Worcester, Massachusetts, United States of America; 3 Cummings School of Veterinary Medicine at Tufts University, North Grafton, Massachusetts, United States of America; Colorado State University, UNITED STATES

## Abstract

The characterization of immortalized canine osteosarcoma (OS) cell lines used for research has historically been based on phenotypic features such as cellular morphology and expression of bone specific markers. With the increasing use of these cell lines to investigate novel therapeutic approaches prior to *in vivo* translation, a much more detailed understanding regarding the genomic landscape of these lines is required to ensure accurate interpretation of findings. Here we report the first whole genome characterization of eight canine OS cell lines, including single nucleotide variants, copy number variants and other structural variants. Many alterations previously characterized in primary canine OS tissue were observed in these cell lines, including *TP53* mutations, *MYC* copy number gains, loss of *CDKN2A*, *PTEN*, *DLG2*, *MAGI2*, and *RB1* and structural variants involving *SETD2*, *DLG2* and *DMD*. These data provide a new framework for understanding how best to incorporate *in vitro* findings generated using these cell lines into the design of future clinical studies involving dogs with spontaneous OS.

## Introduction

Established cell lines are commonly utilized in preclinical cancer research to help dissect many facets of tumor biology, including sensitivity to novel therapeutics and the role of molecular and genetic aberrations in disease progression [1, 2]. The past decade has witnessed unprecedented growth and utilization of tumor genomics data to guide therapeutic, diagnostic, and prognostic approaches. Therefore, continued accurate incorporation of *in vitro* data in cancer research requires a complete understanding of the genomic landscape of these tools. This is particularly relevant in preclinical evaluation of targeted therapeutics, which rely on knowledge of the spectrum of genetic alterations in cancer cells. As such, cell line whole genome and exome sequencing (WGS and WES, respectively) are increasingly evaluated contemporaneously with primary tumor samples [3–13].

While extensive documentation of human and murine tumor cell lines has been performed, canine tumor cell lines have undergone a relatively limited genomic analysis. Given that dogs with spontaneous cancer are increasingly being leveraged to evaluate new therapeutics in the preclinical setting [14] it is important that the companion tools used for *in vitro* studies be thoroughly defined, particularly with respect to genomic lanscape. For example, a variety of established canine osteosarcoma (OS) cell lines are employed in preclinical studies; however, they have been primarily characterized using methods that define a relatively narrow spectrum of molecular and pathway alterations. A limited number of OS lines have been evaluated using RNA-seq and WES, demonstrating conserved transcriptional signatures and point mutations in *TP53* with sequenced tumors [1, 15–17]. We and others have recently characterized canine primary OS using WGS, WES and RNA-sequencing, demonstrating significant structural complexity, including aberrations in *SETD2*, *DMD*, *DLG2* and *MYC*, among others [16–19]. Several of these were not noted in the prior interrogation of canine OS cell lines, largely due to the fact that a defining feature of canine OS is the presence of large structural changes that are more difficult to detect via WES [18–20]. Therefore, we performed WGS in eight canine OS cell lines to characterize the tumor genome landscape and assess the similarities and differences between these cell lines and naturally occurring canine primary OS tumors.

## Materials and methods

### Cell line acquisition and DNA extraction

The OSCA2 and OSCA8 (*e*.*g*. OSA2 and OSA8) cell lines were a generous gift from Dr. Jamie Modiano (University of Minnesota). The Abrams and Gracie cell lines were provided by Dr. Douglas Thamm (Colorado State University). Genomic DNA extracted from the HMPOS, McKinley (*e*.*g*. MacKinley), Moresco (*e*.*g*. Marisco) and OS2.4 cell lines were provided by Dr. Douglas Thamm (Colorado State University). The remaining four cell lines (OSCA2, OSCA8, Abrams, Gracie) were confirmed to be mycoplasma negative by PCR prior to DNA isolation. DNA was isolated using the DNeasy Blood & Tissue Kit (Qiagen Inc., Hilden, Germany). We performed additional cell line validation via short tandem repeat (STR) profiling on the extracted gDNA used for WGS with commercially available loci (Stockmarks canine genotyping kit, Applied Biosystems) per manufacturer recommendations and compared to available published loci for each cell line when available [1].

### Library construction and sequencing

WGS was performed by the Broad Institute Genomics Platform on an Illumina platform with sample tracking with automated LIMS as previously described [18]. Briefly, 100 ng of genomic DNA underwent shearing using a Covaris ultra-focused sonicator, followed by SPRI bead cleanup. The KAPA Hyper Prep Kit with Library Amplification Primer Mix (KAPA Biosystems; #KK8504) was used with palindromic forked adaptors containing a unique 8-base index sequence (Roche). Following normalization of libraries to 2.2nM, cluster amplification and sequencing was completed on a HiSeqX, utilizing Sequencing-by-Synthesis Kits to generate 151-bp paired-end reads. Samples were sequenced to a target depth of 30x.

### Preprocessing of sequencing data

**Fig 1:**
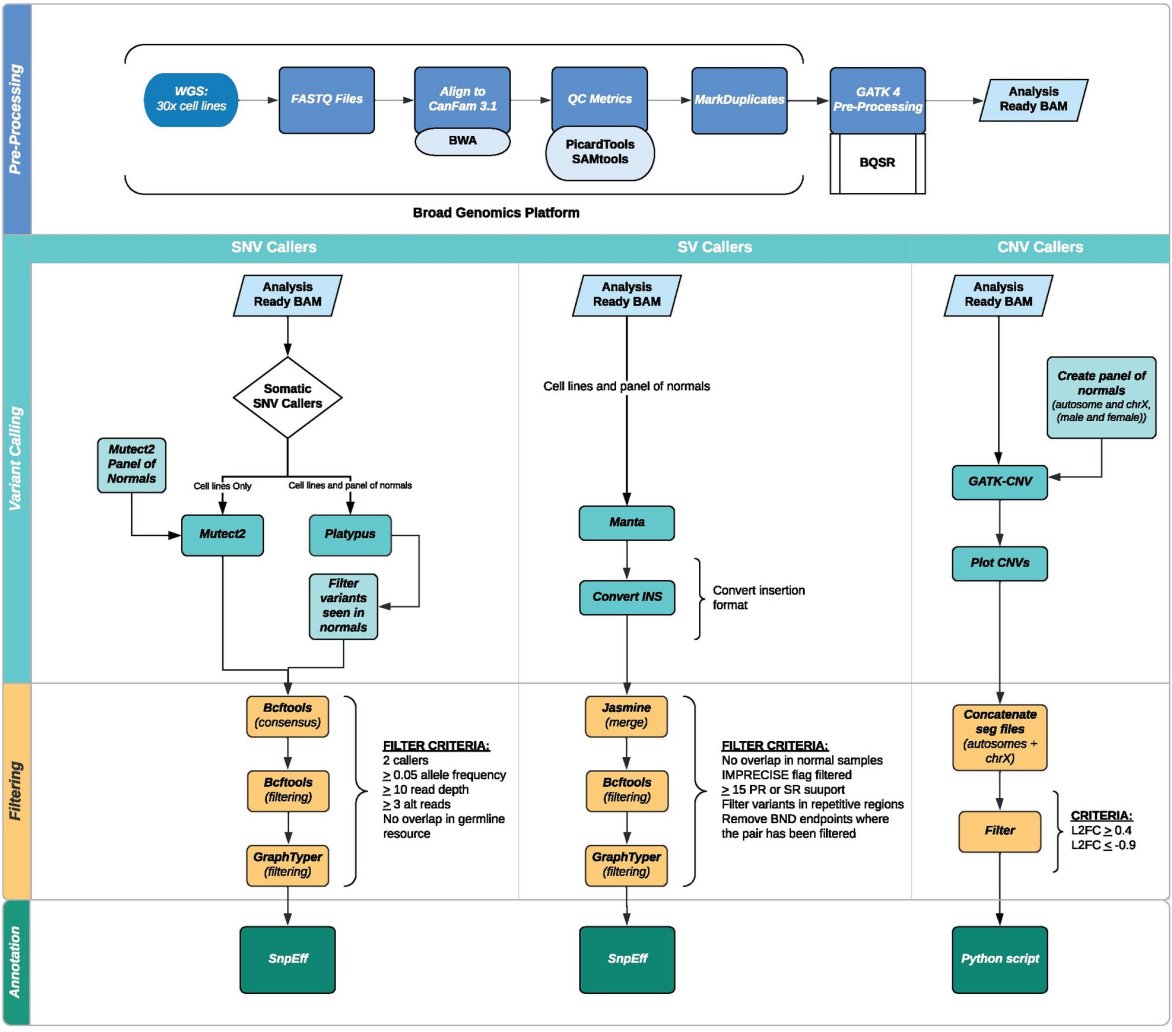
Data analysis pipeline. Overview of analytic approach.

Samples achieved a mean sequencing depth of 53.7x (range 38.5x - 80.9x, S1 Table). Cell line sequencing data was processed through the workflow illustrated in Fig 1. Briefly, fastq files were aligned to the canine reference genome (CanFam3.1 [21]) using BWA [22] and subsequently underwent quality control following the GATK best practices [23, 24]. For all GATK tools, version 4.1.3.0 was used, unless otherwise stated. Duplicate reads were identified using Picard Tools MarkDuplicates (http://broadinstitute.github.io/picard). Base Quality Score Recalibration (BQSR) was performed using a VCF file containing germline variants identified in 676 dogs and other canids [25, 26].

### Simple somatic mutation calling

Simple somatic mutations (single nucleotide variants (SNVs) and small insertions/deletions (indels)) were detected using a consensus calling approach combining Mutect2 and Platypus, both of which permit variant calling without a matched normal sample [27, 28] (S1 Fig). Mutect2 was run using the GATK Showcase WDL scripts available on the Terra cloud computing platform. First, a panel of normal variants was generated using germline WGS data from 23 dogs from a previously published dataset [18]. The VCF of germline variants called in 676 dogs and other canids was used as a germline reference, and a subset of these variants were used in the CalculateContamination step. Mutect2 was run with the additional arguments “—downsampling-stride 20—max-reads-per-alignment-start 6—max-suspicious-reads-per-alignment-start 6.” FilterMutectCalls was run with the “—run_orientation_bias_mixture_model_filter” option set to “True” and the “—min-median-read-position" option set to 10 bp. The unanchored and mitochondrial chromosomes were excluded from variant calling. Variants were also called on the cell lines and 23 normal published WGS BAMs [18] using Platypus (v. 0.8.1), with the “—minReads” flag set to 3.

We employed a multi-step filtering process to identify high-confidence variant calls and eliminate putative germline variants to the extent possible. As our germline reference VCF had been updated to remove two individuals and all variants not supported by the remaining 674 individuals, we updated the filter flag in the Mutect2 calls to reflect these changes. Step 1: using Bcftools (v. 1.12) [29] the filter flag was reset to “PASS” for any variant in the Mutect2 output overlapping the position of a variant removed from the germline reference and where the filter field was set to “germline” only. Step 2: the same approach was used to reset the “alleleBias” flag in the Platypus output, as this could remove low allelic fraction somatic variants. Step 3: a panel of normals was created for the Platypus data by merging the variant calls from the same germline samples used in the Mutect2 panel. The Bcftools isec command was then used to remove variant calls in the Platypus cell line data that overlapped with the position of a variant called in the panel of normals. Step 4: sites with a non-passing filter flag were removed using Bcftools view. Step 5: Bcftools isec was used to keep only variants called in both Mutect2 and Platypus for each cell line. Step 6: Bcftools isec was used to remove putative germline variants seen in the germline reference VCF, in the set of Broad germline SNPs [30, 31], or in the set of Axelsson germline SNPs [32]. Step 7: Bcftools view was used to remove variant calls with an allelic fraction (AF) < 0.05, read depth (DP) < 10, or fewer than 3 reads supporting the alternate allele. Step 8: remaining putative somatic sites were regenotyped in 23 normal germline samples using the Graphtyper [33] tool, and variants found in the germline samples were filtered out using Bcftools. Passing variants were annotated using SnpEff v5.0e [34]. The KaryoploteR package, using R (R3.5.0) was implemented to identify areas of kataegis [35]. Lollipop mutation plots were created using the lollipops tool [36]. Recurrently mutated genes were prioritized for probable relevance in canine OS as previously described [18].

### Breed calling

The preprocessed BAM files were genotyped at putative germline variant locations using GATK HaplotypeCaller (version 4.1.0.0) with the setting—genotyping-mode GENOTYPE_GIVEN_ALLELES. An earlier version of our germline reference was used as the list of sites to be genotyped. This germline reference contained 435 samples (287 pure breed dogs, 6 dogs with unknown ancestry, 100 worldwide indigenous or village dogs, 36 wolves, and 6 other wild canids). To determine the breed of each cell line, the breed calling pipeline was created by selecting publicly available genotype data (N = 1,212) [25, 26, 37] from 101 modern breeds with at least 12 purebred dogs per breed. Wright’s F-statistics using Hudson method was calculated for each breed using 2,468,442 biallelic single nucleotide polymorphisms with <10% missing genotypes. SNPs with FST>0.15 across all comparisons were selected and LD-based pruning in 50kb windows (r2>0.5) was performed to extract 688,060 markers for global ancestry inference. We merged genotypes for these SNPs from the cell lines with genotypes from reference samples, then performed global ancestry inference using ADMIXTURE [38] in supervised mode (random seed: 43) [26].

### Mutational signature calling

The SigProfilerMatrixGenerator tool [39] was used to generate a matrix of variant mutational contexts. We then used the SigFit tool (v2.2) [40] to identify the COSMIC v3 [41] single base substitution (SBS) signatures present in the cell line data. The mutational opportunities matrix for the CanFam3.1 genome was kindly provided by Adrian Baez-Ortega, University of Cambridge, one of SigFit’s authors. Fitting was run with 10000 iterations and 5000 warmup iterations, using the multinomial model. Signatures that were sufficiently greater than zero (meaning that the lower end of the Bayesian HPD interval was > 0.025 in any sample) were selected and fitting was rerun using only those signatures.

### Structural variant calling

Somatic copy number aberrations (SCNAs) were detected using the GATK somatic CNV pipeline [24, 42], via the Terra showcase workspace WDLs (Fig 1). An autosomal panel of normals was created using all 23 germline samples, and male-only and female-only panels were created for chromosome X. The “do_explicit_gc_correction” option was set to “True” for panel creation. As the sex of the donor was not annotated for many of the cell lines, we determined the sex based on the ratio of average read depths across the autosomes and chromosome X (as determined by the GATK DepthOfCoverage tool). Ratios of X/autosome coverage between 0.3 and 0.7 were considered male, and ratios between 0.8 and 1.2 were considered female. CNV calling was performed, with smoothing parameters “kernel_variance_allele_fraction” and “kernel_variance_copy_ratio” set to 0.8, and “num_changepoints_penalty_factor” set to 5. CNV plots were remade using a sorted DICT file to plot the chromosomes in numeric order and exclude the unanchored and mitochondrial chromosomes. Copy number losses with a log_2_ fold change of ≥ 0.4 (one copy gain) or ≤ -0.9 (two copy loss) were considered in our analysis. A custom Python script was used to annotate the overlap of copy number segments with genes using the Ensembl canine gene annotation (Release 99) [43].

Structural variants (SVs) were called using Manta version 1.6.0 [44]. Cell lines and the 23 normal germline samples were run separately using settings for tumor-only or germline as appropriate. The output VCFs were processed using the Manta-provided script “convertInversion.py” to convert inversions to the older INV format, rather than the current break end (BND) format. To mitigate the incidence of false positives when analyzing unmatched tumor-derived samples, multiple filtering steps were performed. Step 1: a panel of normals was created by merging SV calls for the 23 germline VCFs with each of the cell line VCFs using the Jasmine tool [45], using the “—nonlinear_dist max_dist = 1000”, "—output_genotypes”, and “—keep_var_ids” settings. A custom Python script was used to add genotypes to the “GT” field so that the VCFs could be parsed by Bcftools. All genotypes were set to 0/1. For each cell line-panel of normals merged VCF, the variant IDs of variants present in the cell line but none of the normals were extracted. Step 3: using Bcftools, variant IDs present in the normals were removed, as well as variants where the filter field was not “PASS”, that were flagged as “IMPRECISE”, or where neither the paired read (PR) nor split read (SR) support was greater than or equal to 15. The Jvarkit “vcfbedsetfilter” tool [46] was used to flag variants overlapping putative centromeric regions (5000bp windows containing ≥80% centromeric repeats, from https://github.com/Chao912/Mischka/CanFam3.1.centromere.bed). Step 4: remaining unfiltered variants were regenotyped in the normal samples using the Graphtyper tool [33], and any variant with support in a normal sample was removed using Bcftools. Step 5: translocation break ends where one end had been filtered out in a previous step were removed using Bcftools.

### Comparison to literature

We identified five published WES or WGS datasets of canine OS tissue (Sakthikumar, *et al*. [16], Gardner, *et al*. [18], Das, *et al*. [17], Chu, *et al*. [19]) or cell lines (Das, *et al*. [1]). Variant calls were obtained in VCF or tabular format from supplementary data and standardized into VCF format. To minimize variability due to gene annotation and sequencing strategy, we limited our comparison to coding regions (specifically, the CDS regions in the Ensembl canine annotation Release 99) using Bcftools view, and reannotated the VCFs from each study using Snpeff. Variants annotated as low impact were excluded. Structural variants, including copy number variants from Gardner, *et al*. and Chu, *et al*., were converted from tabular format into bed files. Overlapping regions within each sample were merged using Bedtools merge [47]. Copy number segments found to be significantly recurrently altered in Sakthikumar, *et al*. were also converted into bed format for comparison, but no sample-level CNV count could be performed. Genes overlapped by a structural variant were annotated by using Bedtools annotate to count the number of overlaps of the CDS regions in the Ensembl canine annotation within each dataset. Due to lack of reported breakpoint end coordinates for translocations in the literature no standardization could be done, and translocations were compared by counting the number of times a given gene was annotated as affected in each dataset.

## Results

### Cell line validation

DNA isolated from each cell line was extracted and confirmed to be of canine origin and the stated cell line of origin via multi-platform interrogation. STR profiling and species-specific PCR confirmed sequenced DNA was canine, and STR loci were consistent with those previously reported [1] (S2 Table). Additionally, breed-calling and sequencing coverage over the X chromosome confirmed the breed and sex origin of the tumor cell lines when previously published data were available, and identified this information for several lines in which that information was not publicly available (S3 Table). Importantly, village dogs do not have breed ancestry, resulting in the breed calling algorithm calling many different breeds, each reported as contributing a small fraction. This becomes particularly important for WGS datasets where a germline DNA reference sample is not available, as existing databases of germline variation may not accurately capture the spectrum of normal germline variants in these dogs, resulting in the spurious appearance of a higher mutation burden. Finally, single nucleotide variant (SNV) calls among the different cell lines were not concordant, consistent with the cell lines being properly identified and no cross-contamination occurring between cell lines.

### Single nucleotide variants in canine OS cell lines

**Fig 2:**
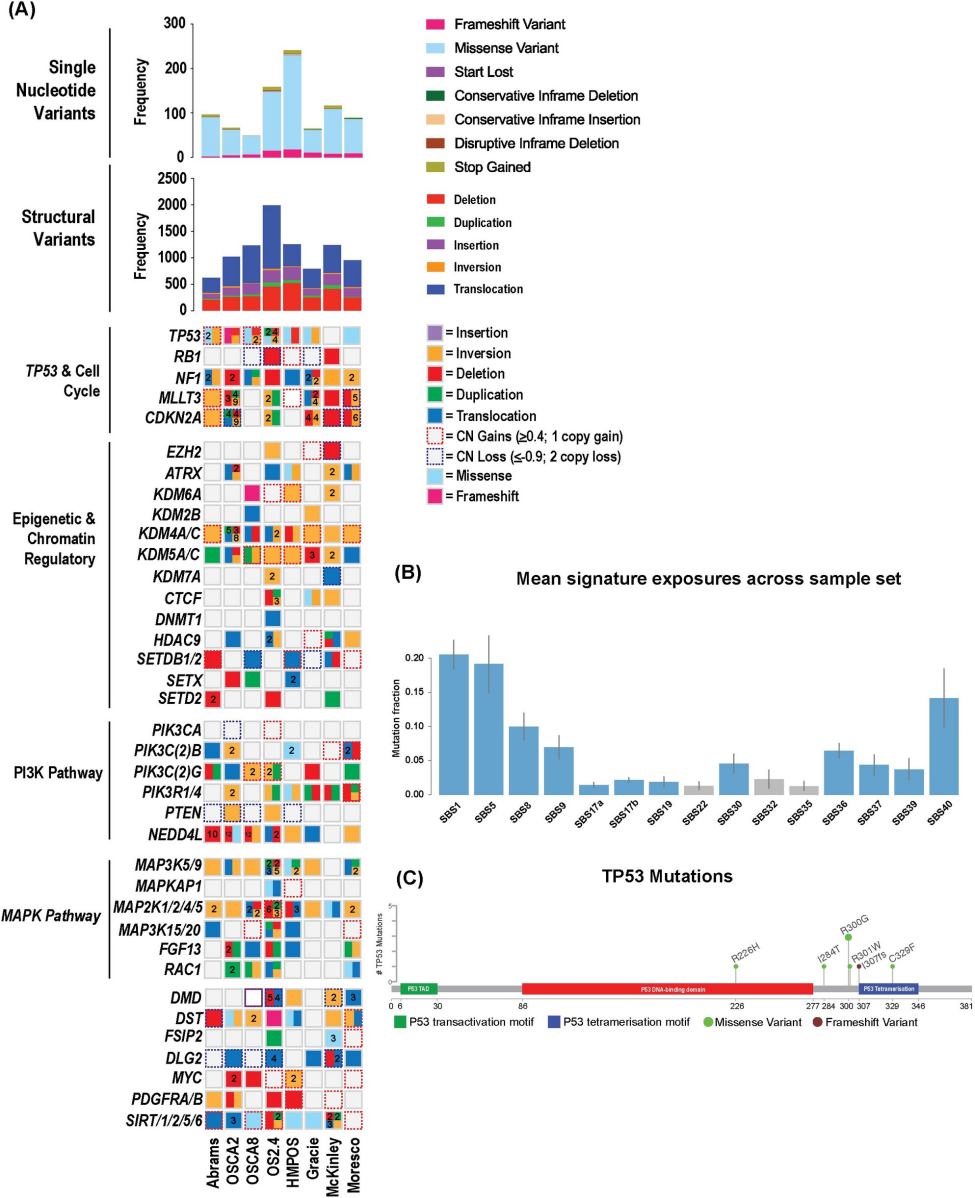
Mutation landscape in canine OS cell lines. (A) Oncoprint illustrating key mutations and copy number aberrations in canine OS cell lines. (B) Summary of common mutational signatures present in canine OS cell lines. Error bars indicate the 95% highest posterior density (HPD) intervals. Blue bars represent signatures with mean exposure of 0.01 or higher in the cohort; grey bars had a mean exposure < 0.01 in the cohort, but a lower value of the HPD interval ≥ 0.025 in at least one cell line. (C) Lollipop plot characterizing the SNVs identified in *TP53*.

**Fig 3:**
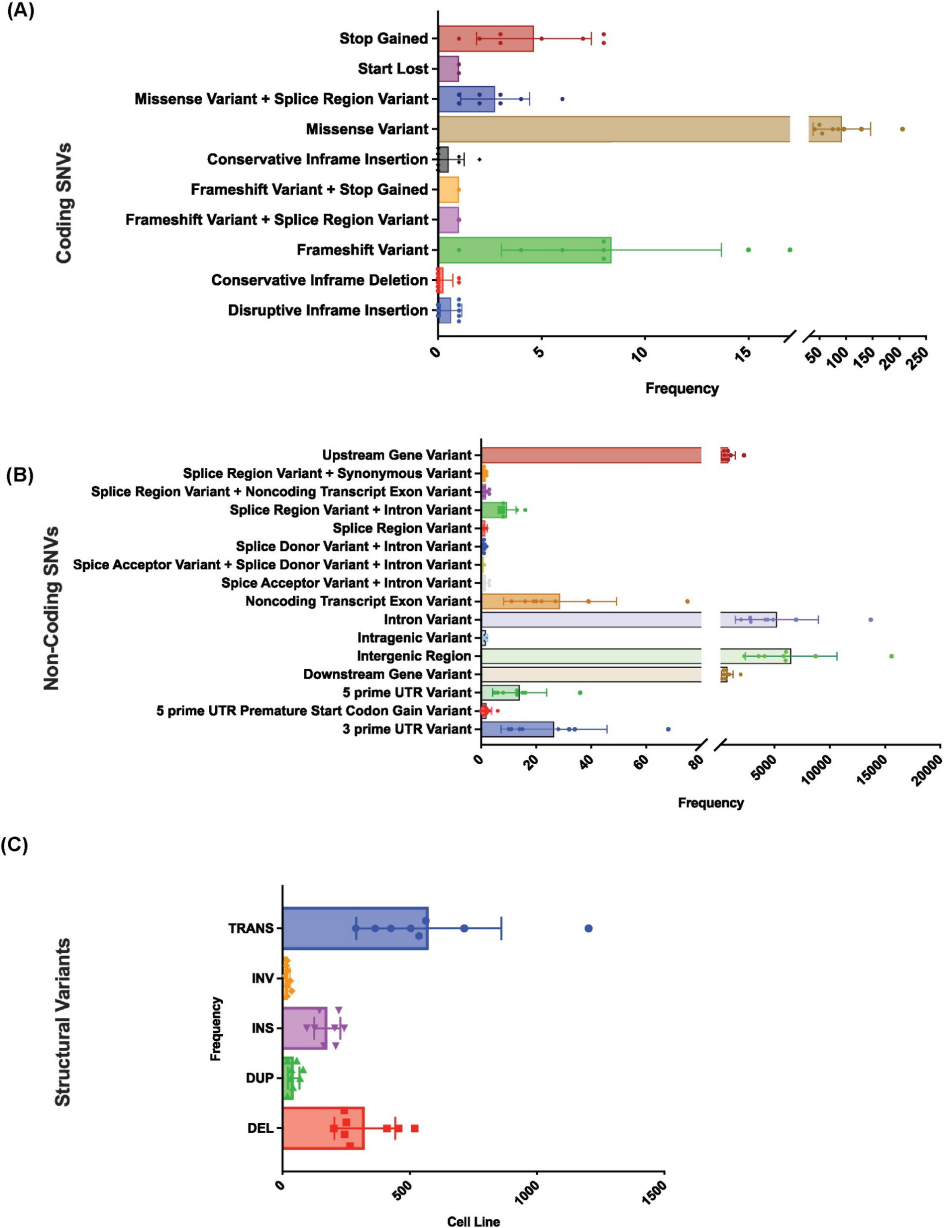
Frequency of SNV and SV calls in OS cell lines. Frequency of (A) coding and (B) noncoding SNVs across canine OS cell lines. (C) Frequency of structural variants across canine OS cell lines. Bars represent mean with individual data points. Error bars represent standard deviation.

Missense mutations were the most common coding SNV identified in canine OS cell lines, with a smaller fraction of frameshift mutations and other disruptive events (Figs 2 and 3A, S4 Table). Not surprisingly, a high incidence of noncoding variants including splice region variants, 3’ and 5’ untranslated region variants were identified (Fig 3B). It is likely that the lack of a matched germline reference led to a higher incidence of false positive calls in the noncoding genome. However, variants in regulatory regions are increasingly recognized as contributing to tumorigenesis [48]. While the significance of these variants is unknown, further interrogation of noncoding mutations that can affect cancer driver genes is warranted to begin attributing functional significance to noncoding elements in OS.

**Fig 4:**
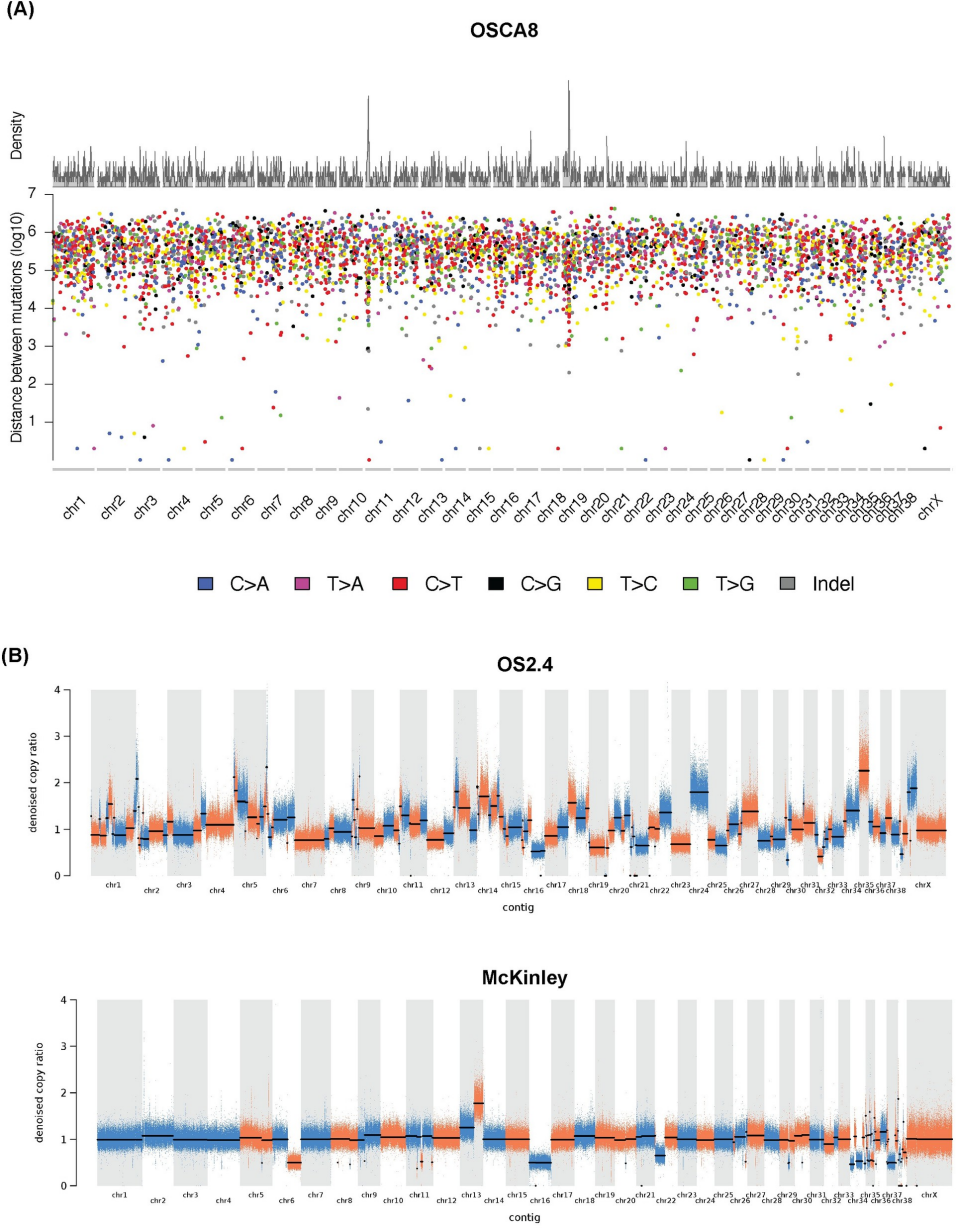
OS cell lines exhibit regions of focal hypermutation and multiple copy number aberrations. (A) Rainfall plot demonstrating regions of focal hypermutation in the OSCA8 cell line. (B) Copy number ratio plots, demonstrating the presence of both focal and whole chromosome level copy number aberrations in the OS2.4 and McKinley cell lines. Copy ratio segments are highlighted alternating between blue and orange, while the denoised median is represented by the black lines.

Despite extensive filtering, the mutational burden in each cell line, calculated at 5.8 mut/Mb (range 2.1–14.7, S5 Table) was higher than previously reported in canine and human primary OS tissues [18, 49]. This is likely a result of long-term passaging of cell lines and lack of a germline reference sample from the individual in which the tumor originated. In the Gracie and OSCA-8 cell lines, regions of focal hypermutation suggestive of kaetegis were identified (Fig 4A, S3 Fig). The HMPOS cell line, which originated in a village dog whose ancestry is not well represented in our reference panel, had the highest apparent mutational burden.

Consistent with primary OS tissue samples, the most common coding SNVs were mutations in *TP53* (7/8; 88%), predominantly made up of missense mutations with a smaller incidence of frameshift mutations (Fig 2A and 2C). The only other gene with coding SNVs identified in at least three cell lines was *DST*, a gene encoding dystonin, a cytoskeletal linker protein. All other recurrent coding SNVs were private to one or two cell lines. However, the spectrum of SNVs observed was largely representative of that identified in primary canine OS tissue samples, with mutations involved in DNA repair and the cell cycle, epigenetic and chromatin regulatory genes, and PI3K and MAPK signaling pathways.

We compared our simple somatic mutation calls to those previously reported in WES/WGS canine OS tissue samples (S6 Table) [16–19]. Of the 3836 genes with SNVs/INDELs reported in at least one OS tumor in these studies, 272 (7%) were also mutated in at least one cell line. *TP53* was the most commonly mutated, both in the literature (64%) and in our data (88%). Of the genes reported in at least 5% of OS samples, *FSIP2* (13% cell lines, 11% reported in the literature), *TTN* (3% cell lines, 9% reported in the literature), *ENSCAFG00000000632* (13% cell lines, 7% reported in the literature), *RYR2* (13% cell lines, 5% reported in the literature), *UNC80* (13% cell lines, 5% reported in the literature), *LRP1B* (13% cell lines, 5% reported in the literature), and *XIRP2* (13% cell lines, 5% reported in the literature) were mutated in at least one cell line. Several genes commonly reported as mutated in OS samples did not have simple somatic mutations in any of the cell lines, most notably *SETD2* (19% reported in the literature), as well as *NEB* (12% reported in the literature). We also examined the concordance of our SNV and INDEL WGS calls those previously reported from WES sequencing of the same cell lines (S6 Table) [1]. Overall, an average of 49% of coding variants reported in WES of these cell lines were confirmed by WGS (range 35% (McKinley)– 73% (OS2.4)).

The trinucleotide context of SNVs was evaluated, identifying exposure to the COSMIC v3 single base substitution (SBS) signatures in the SNV calls from the cell lines. Signatures SBS1 (the “aging signature,” associated with spontaneous deamination of 5-methyl-cytosine), SBS5 (a “clock-like” signature of unknown etiology), SBS8 (unknown etiology), SBS9 (possibly due to somatic hypermutation via polymerase eta in lymphoid cells), SBS17a (unknown etiology), SBS17b (associated in some human cases with fluorouracil chemotherapy and reactive oxygen species damage), SBS19 (unknown etiology), SBS22 (aristolochic acid exposure), SBS30 (base excision repair deficiency, associated with loss of NTHL1 function), SBS32 (associated with azothiaprine treatment), SBS35 (associated with platinum-based chemotherapy), SBS36 (base excision repair deficiency, associated with loss of MUTYH function), SBS37 (unknown etiology), SBS39 (unknown etiology), and SBS40 (etiology unknown, associated with aging in some human cancers) were identified in varying proportions across the cell lines [41]. The highest contributions were made by signatures SBS1, SBS40, and SBS5 (Fig 2B, S4 Fig). Signatures SBS1, SBS5, SBS8, SBS17a, SBS17b, SBS30, and SBS40 have previously been reported in human OS samples [50], while signatures SBS1, SBS8, SBS9, and SBS17b have been reported in canine OS [16–19].

### Structural variants in canine OS cell lines

SVs, including deletions, insertions, inversions, translocations, and duplications were identified (S7 Table). The average incidence of SVs in this panel of cell lines was 1139 SVs per cell line, which is markedly higher than reported in OS tissues [18] and likely a result of the lack of an available matched germline sample. The most common SVs were deletions and complex chromosomal translocations (Figs 2A and 3C). Consistent with SVs reported in primary OS tissues, non-copy number structural variants involving *DMD* (4/8 (50%) cell lines), *DLG2* (5/8 (62.5%) in this study), *CDKN2A* (6/8 (75%)), *MAGI2* (7/8 (88%)), and *MLLT3* (6/8 (75%)) were present (S7 Table). Notably, multiple variants involving epigenetic and chromatin regulatory genes were identified in all cell lines, supporting previous assertions implicating alterations of the epigenetic landscape in OS biology [51, 52]. Large-scale deletions spanning *SETD2* were found in 2/8 (25%) of cell lines in this study, while one other cell line had a duplication involving *SETD2*. Finally, additional recurrent SVs were identified in *NF1* (8/8 (100%)), *NEDD4L*, an E3 ubiquitin ligase responsible for *PTEN* homeostasis (7/8 (88%)), as well as in histone demethylase genes *KDM4A* and *KDM4C* (alteration in one of the two present in all cell lines in this study), and *KDM5A* and *KDM5C*, (alteration in one of the two present in all cell lines in this study).

### Somatic copy number aberrations

Both focal and chromosome-level somatic copy number aberrations (SCNAs) were identified in all OS cell lines (S5 Fig, S8 Table). We compared our results to SCNAs reported in the literature. Many of the most common copy losses (log2 fold change ≤ -0.9) have also been commonly reported in OS tumors, including exonic *DLG2* (50% in this study, 28% in prior literature), *CDKN2A/B* (38% in this study, 44% in prior literature), *PTEN* (50% in this study, 44% in prior literature), and *MAGI2* (38% in this study, 36% in prior literature) (S6 Table). In addition, copy losses were present in the classic tumor suppressor gene *RB1* (38% in this study, 8% in prior literature) and the recently reported *DMD* (38% in this study, 36% in prior literature) (Fig 2A) [18]. Similarly, recurrent copy number gains (log2 fold change ≥ 0.4) were present in *MYC* (38% in this study, 36% in prior literature), consistent with the reported incidence in primary canine OS tissues (Fig 2A, S8 Table).

### Preservation of epigenetic pathway aberrations in OS cell lines

While many mutations and SCNAs in OS-associated genes, such as *TP53*, *MYC*, *CDKN2A/B* and *DLG2*, were preserved, some mutations previously identified at a high incidence were absent in the cell lines evaluated. In particular, no SNVs were identified in *SETD2* in the eight OS cell lines evaluated. However, mutations in histone 3 lysine 36 (H3K36) specific lysine demethylase genes (*KDM2B*, *KDM4A*, *KMD4C*, *KDM7A*) were present in all cell lines (Fig 2A, S9 Table). As the biologic activity of SETD2 is thought to be due to its effect on H3K36 trimethylation, these data suggest that curation of mutations leading to H3K36 dysregulation may be more relevant in OS. Notably, the most commonly amplified region across all cell lines was a segment of chromosome 35 containing numerous histone proteins.

Similarly, a variety of mutations and copy number aberrations were identified in PI3K and MAPK pathway genes (Fig 2A, S4 and S8 Tables), with all cell lines having at least one alteration in *MAP2K1*, *MAP2K2*, *MAP2K4*, or *MAP2K5*. Consistent with the notion that OS is genomically heterogenous, few aberrations in individual genes were recurrent. Additionally, copy number losses, deletions, inversions, and translocations were identified in *PTEN* (5/8 (62.5%) cell lines) and *NEDD4L* (7/8 (88%)) (Fig 2A, S7 and S8 Tables), suggesting that PI3K pathway dysregulation mediated by PTEN should be considered in the context of concurrent mutations in *NEDD4L*, an E3 ubiquitin ligase that negatively regulates PTEN.

## Discussion

Established cell lines have long been used to study tumor biology and response to targeted therapies. More recently, single gene evaluation and WES have been used to chart the mutational landscape of canine cancer cell lines, providing a crucial resource for prospective studies [1, 53–70]. The WGS data reported here identified many simple somatic mutations previously published in WES datasets. However, the use of WGS permitted interrogation of CNVs and SVs, enabling a more complete understanding of the spectrum of pathway dysregulation in canine OS cells. This is particularly important in genomically complex cancers, such as OS, where hotspot SNVs are less common.

While many of the known simple somatic mutations associated with canine OS were conserved among the cell lines evaluated in this study, some mutations typically found in primary OS tissues were absent. One striking feature was the lack of simple somatic mutations in *SETD2* in the cell lines used in this study. However, *SETD2* was deleted in two cell lines, and mutations in H3K36 lysine demethylases were present, suggesting that mechanisms driving H3K36 dysregulation are a fundamental feature of canine OS. Concordance with SNV/INDEL calls between the same cell lines included in our analysis and the WES analysis by Das, *et al*. was moderate, and the discrepancies noted were likely due to several factors. Different sequencing and variant calling methods are known to have low concordance. In addition, the use of distinct variant filtering thresholds and different germline databases likely resulted in the removal of divergent sets of mutations. Additionally, the selective pressures of *in vitro* culture and ongoing genomic instability typically drive the development of significant genetic heterogeneity between different strains of the same cell line [71, 72].

An increased mutational burden was identified in OS cell lines compared to OS tissues. In part, this likely represents a type I error due to lack of a matched germline sample. This is especially relevant in the HMPOS cell line, which was determined to originate from a village dog based on our breed calling algorithm and has a variety of single nucleotide polymorphisms not cataloged in our germline variant resource files. As most genetic variants are rare [73], and village dogs are more genomically diverse than pure bred dogs [74], lack of a matched normal likely resulted in the highest number of false positive somatic variant calls in the HMPOS line.

Incorporation of a matched germline control is commonly used to minimize false positive mutation calling in WES and WGS datasets. We developed a stringent filtering pipeline for both simple somatic and structural variants to reduce the occurrence of false positives due to the lack of a matched germline sample (S1 and S2 Figs). The foundations of this pipeline are established methods in the field; however, it was applied more stringently in this setting. For example, we removed any variant which overlapped a variant in our germline resource rather than requiring that it be seen in two or more individuals. We did not require the alternate alleles to match, as we found cases where the alleles were noted differently by different tools, despite appearing to be the same variant. Furthermore, we added a regenotyping step with the GraphTyper tool, which identified any support for putative somatic variants in our panel of normals. This step was particularly helpful in filtering out INDELs where different tools might place the start and end positions in alternate locations. We believe this step may account for some of the discrepancies in the simple somatic calls reported in our study and the Das, *et al*. study for the same cell lines. Nevertheless, due to the above challenges and lack of orthogonal validation of our variant calls, we recommend that researchers validate variants of interest with low allelic fraction prior to additional downstream analysis.

Overall, our data demonstrate that the chaotic genomic landscape of canine OS cell lines is concordant with that observed in primary canine OS tumor tissue, defined by high structural complexity and few recurrent point mutations. It is not surprising that some of the common SNVs and SVs found in OS tumor tissue were not identified in this small subset of cell lines, likely due to evolution of the cell lines over time in culture. Perhaps most notably, conservation of mutations in pathways with redundant functional relevance underscores the probable biologic importance of these aberrations in OS. This study highlights important features of each of these cell lines, creating a roadmap for researchers pursuing hypothesis driven precision medicine research.

Last, we have detailed the use of specific tools and modified scripts in this manuscript to facilitate implementation of this pipeline in other canine WES/WGS datasets in which matched germline reference samples are not available. Additionally, as minor changes in versioning and run parameters for computational tools can markedly alter outputs, we have made available our methodologies to facilitate future use of this approach in other canine sequencing datasets.

## Conclusions

Canine OS cell lines are largely representative of the genomic landscape of primary canine OS tissues. Evaluation of the genomic landscape, including structural variation, is important to accurately identify pathway dysregulation in complex cancers when using cell lines in research.

## Supporting information

S1 Fig SNV calling pipeline. Detailed SNV calling pipeline.
(TIF)

S2 Fig SV calling pipeline. Detailed SV calling pipeline.
(TIF)

S3 Fig Rainfall plots. Rainfall plots for each cell line, with associated density plots demonstrating distance between mutations on a log10 scale.
(PDF)

S4 Fig Mutation signatures. Mutation signatures and signature composition of each cell line.
(PDF)

S5 Fig Copy number segmentation. Denoised copy number segmentation plots and alternate-allele fraction ratios for each cell line. Copy ratio segments are highlighted alternating between blue and orange, while the denoised median is represented by the black lines.
(PDF)

S1 Table Sequencing metrics. Sequencing metrics for each cell line.
(XLSX)

S2 Table STR profiling. Results of STR profiling of canine OS cell lines.
(XLSX)

S3 Table Breed calling. Breed calling consensus and sex determination from each cell line.
(XLSX)

S4 Table SNVs. Single nucleotide variants passing filters called in each cell line.
(XLSX)

S5 Table Mutational burden. Mutation burden was determined by dividing the total number of mutations in each cell line and dividing by the combined size of the chromosomes. Total calculated mutation burden (mutations/Mb) is provided for each cell line, as well as the size of each chromosome.
(XLSX)

S6 Table Literature comparisons. Comparison of calls from this dataset to five published OS datasets.
(XLSX)

S7 Table SVs. Structural variants called by Manta in each cell line.
(XLSX)

S8 Table CNVs. Copy number variants and overlapping genes associated with each region called in each cell line.
(XLSX)

S9 Table Curated gene lists. Detailed summary of curated list of structural variants.
(XLSX)
