# Supplementary figures and images for: The genomic landscape of canine osteosarcoma cell lines reveals conserved structural complexity and pathway alterations
Source: PLoS One. 2022 Sep 13;17(9):e0274383. doi: 10.1371/journal.pone.0274383 (PMC9469990; doi:10.1371/journal.pone.0274383)

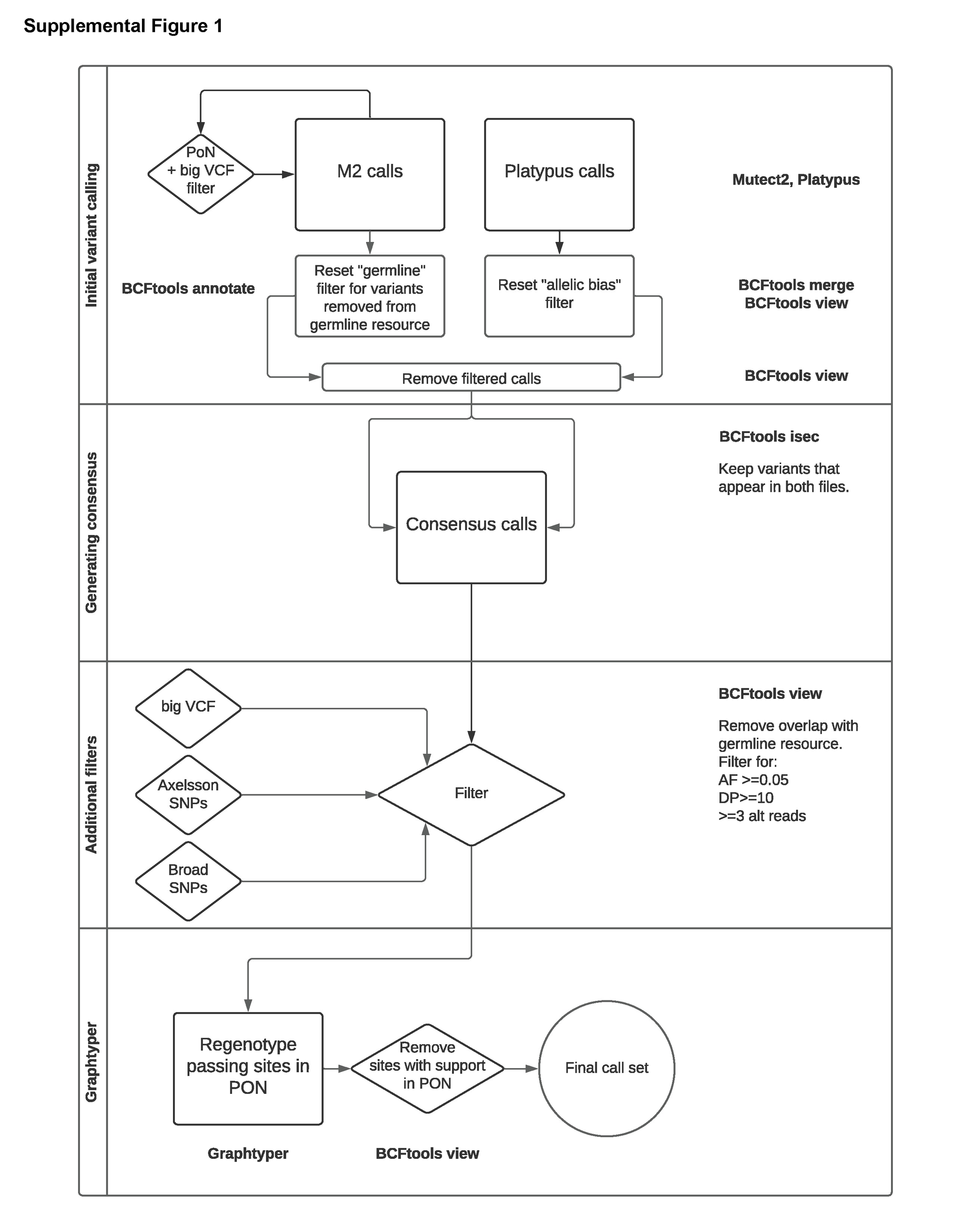

Supplement: S1 Fig — Detailed SNV calling pipeline. (TIF) [file pone.0274383.s001.tif]

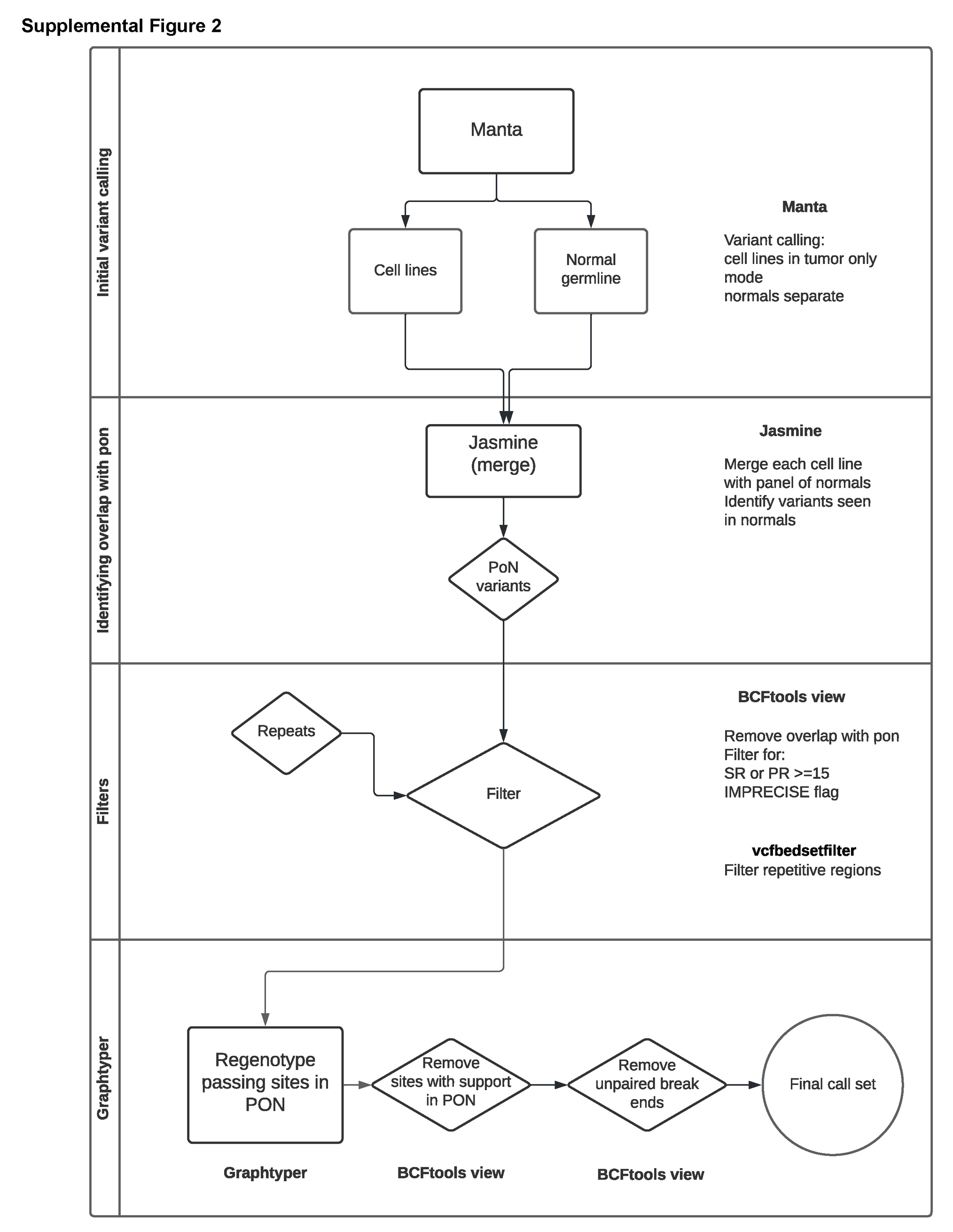

Supplement: S2 Fig — Detailed SV calling pipeline. (TIF) [file pone.0274383.s002.tif]
