# Supplementary material for: The genomic landscape of canine osteosarcoma cell lines reveals conserved structural complexity and pathway alterations
Source: PLoS One. 2022 Sep 13;17(9):e0274383. doi: 10.1371/journal.pone.0274383 (PMC9469990; doi:10.1371/journal.pone.0274383)
Supplement: S3 Fig — Rainfall plots for each cell line, with associated density plots demonstrating distance between mutations on a log10 scale. (PDF) [file pone.0274383.s003.pdf]

Supplemental Figure 3

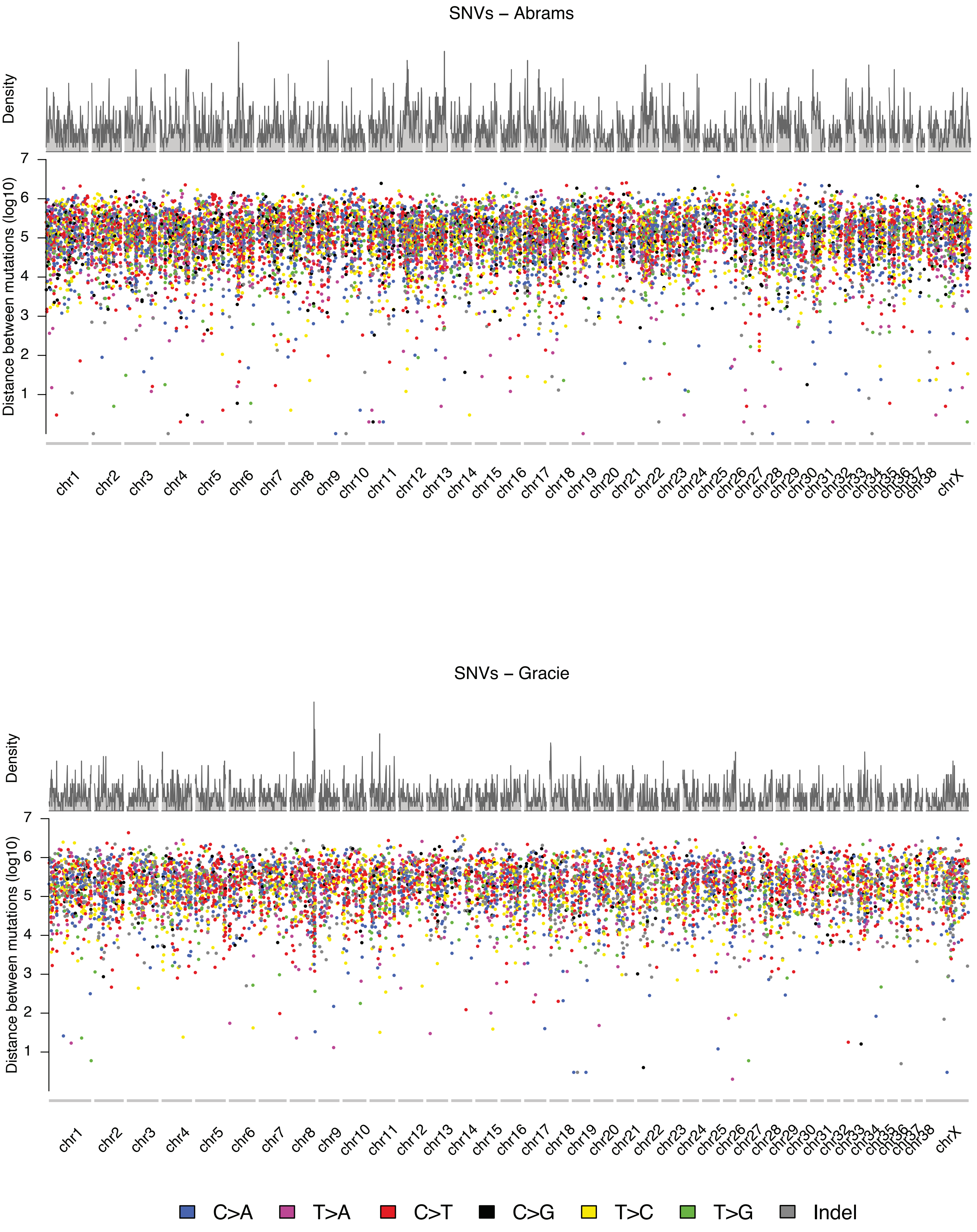

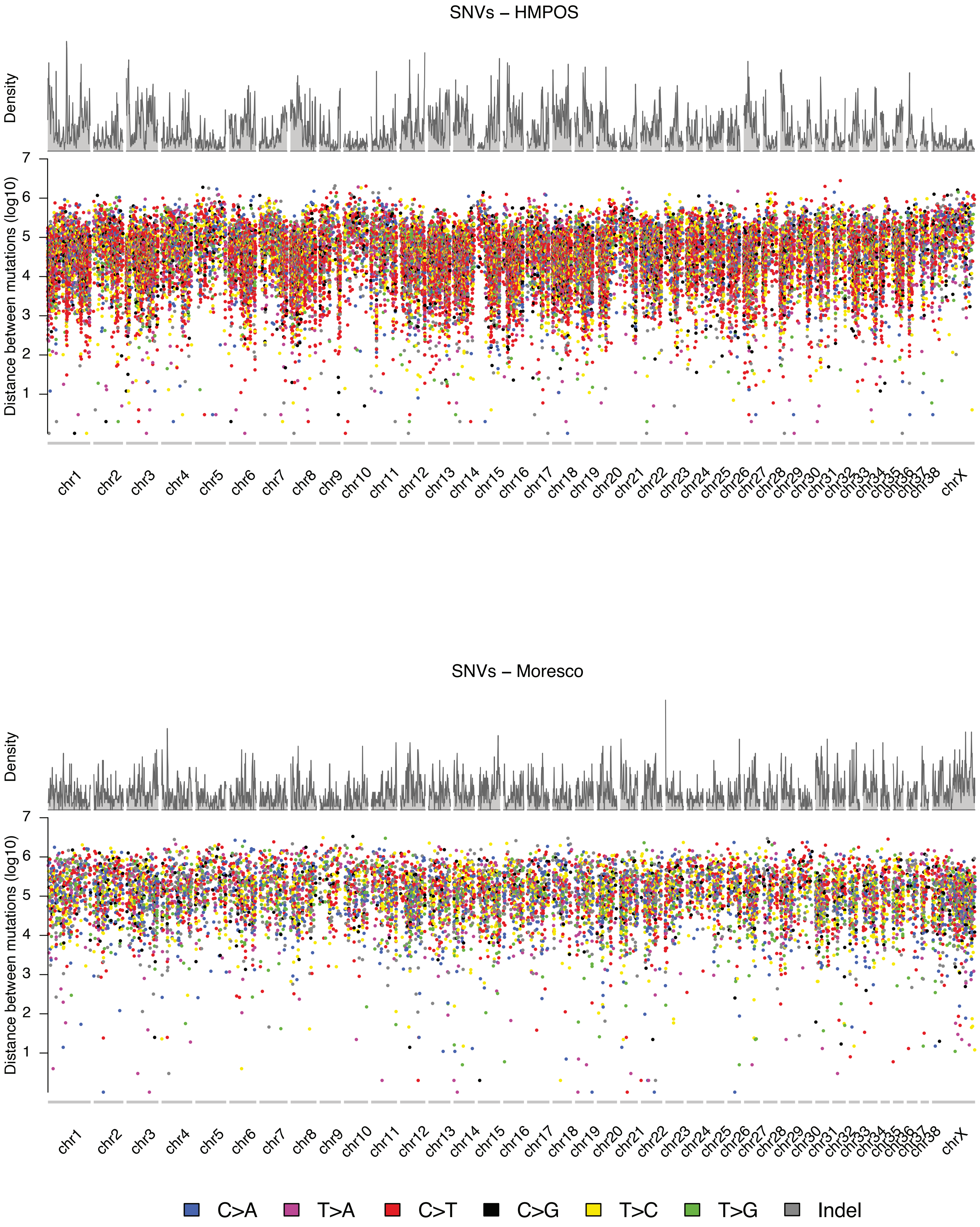
SNVs – HMPOS
Density
Distance between mutations (log10)
1
2
3
4
5
6
7
chr1 chr2 chr3 chr4 chr5 chr6 chr7 chr8 chr9 chr10 chr11 chr12 chr13 chr14 chr15 chr16 chr17 chr18 chr19 chr20 chr21 chr22 chr23 chr24 chr25 chr26 chr27 chr28 chr29 chr30 chr31 chr32 chr33 chr34 chr35 chr36 chr37 chr38 chrX
SNVs – Moresco
Density
Distance between mutations (log10)
1
2
3
4
5
6
7
chr1 chr2 chr3 chr4 chr5 chr6 chr7 chr8 chr9 chr10 chr11 chr12 chr13 chr14 chr15 chr16 chr17 chr18 chr19 chr20 chr21 chr22 chr23 chr24 chr25 chr26 chr27 chr28 chr29 chr30 chr31 chr32 chr33 chr34 chr35 chr36 chr37 chr38 chrX
C>A
T>A
C>T
C>G
T>C
T>G
Indel

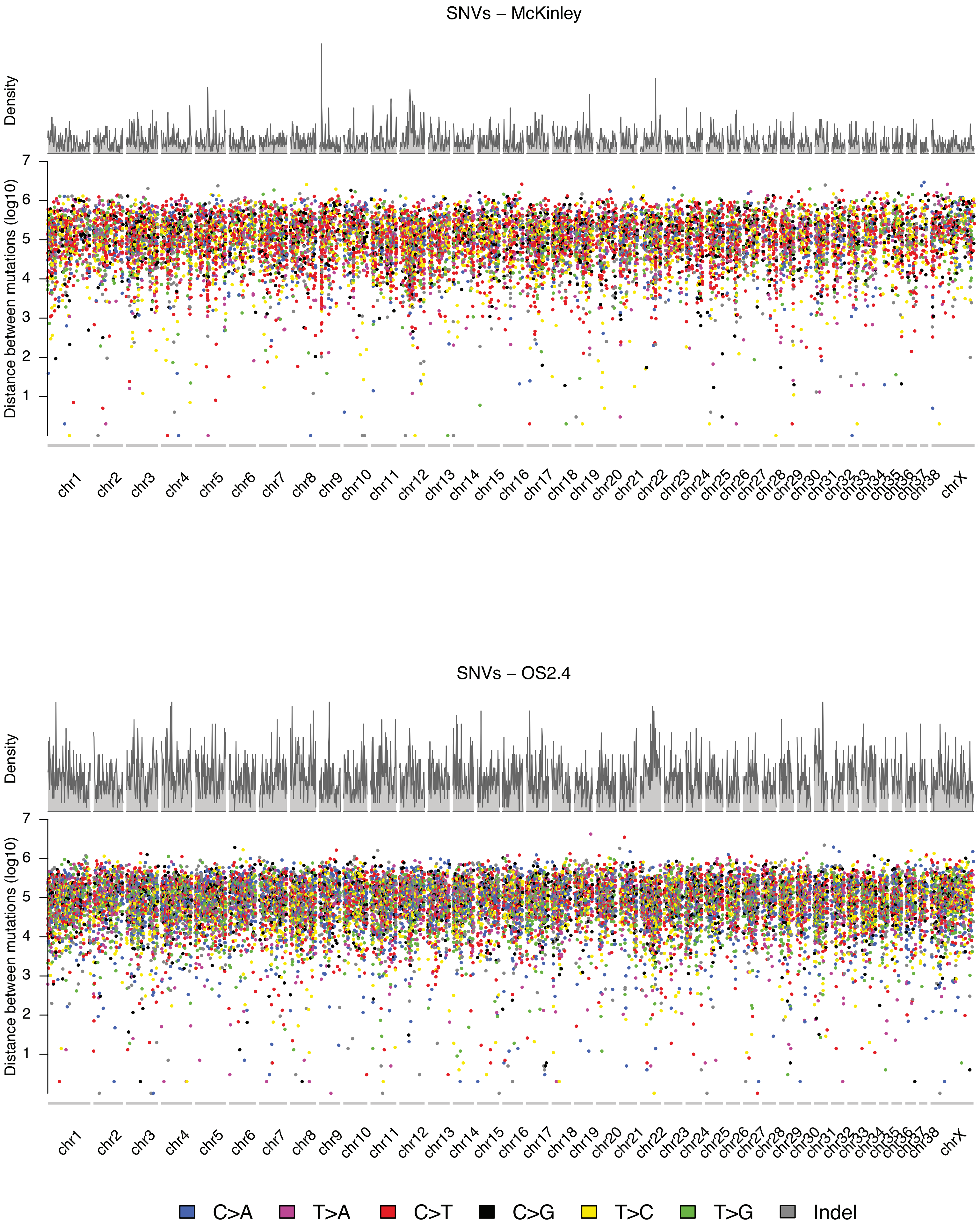
SNVs – McKinley
Density
Distance between mutations (log10)
1
2
3
4
5
6
7
chr1 chr2 chr3 chr4 chr5 chr6 chr7 chr8 chr9 chr10 chr11 chr12 chr13 chr14 chr15 chr16 chr17 chr18 chr19 chr20 chr21 chr22 chr23 chr24 chr25 chr26 chr27 chr28 chr29 chr30 chr31 chr32 chr33 chr34 chr35 chr36 chr37 chr38 chrX
SNVs – OS2.4
Density
Distance between mutations (log10)
1
2
3
4
5
6
7
chr1 chr2 chr3 chr4 chr5 chr6 chr7 chr8 chr9 chr10 chr11 chr12 chr13 chr14 chr15 chr16 chr17 chr18 chr19 chr20 chr21 chr22 chr23 chr24 chr25 chr26 chr27 chr28 chr29 chr30 chr31 chr32 chr33 chr34 chr35 chr36 chr37 chr38 chrX
C>A
T>A
C>T
C>G
T>C
T>G
Indel

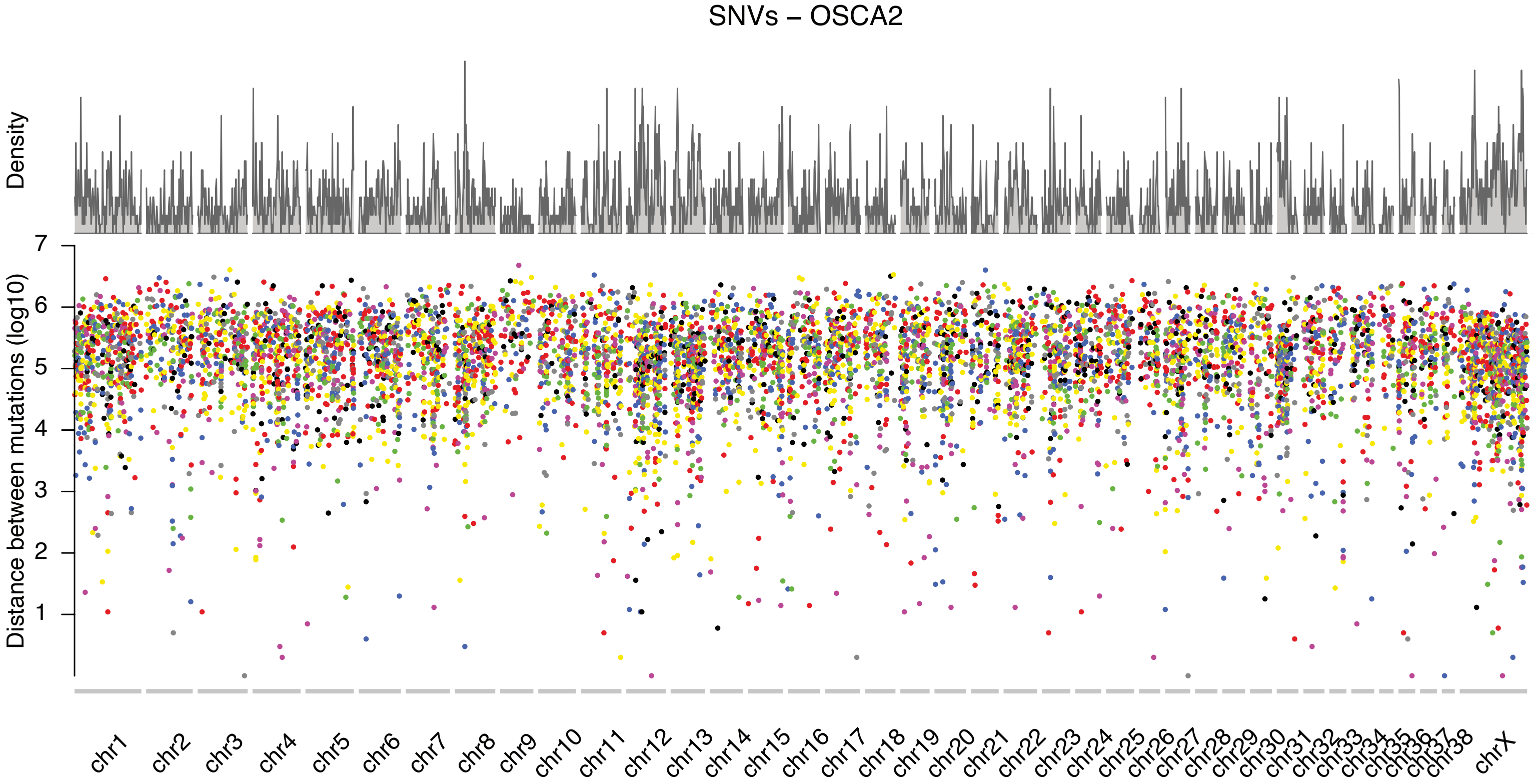
SNVs – OSCA2
Density
Distance between mutations (log10)
1
2
3
4
5
6
7
chr1 chr2 chr3 chr4 chr5 chr6 chr7 chr8 chr9 chr10 chr11 chr12 chr13 chr14 chr15 chr16 chr17 chr18 chr19 chr20 chr21 chr22 chr23 chr24 chr25 chr26 chr27 chr28 chr29 chr30 chr31 chr32 chr33 chr34 chr35 chr36 chr37 chr38 chrX

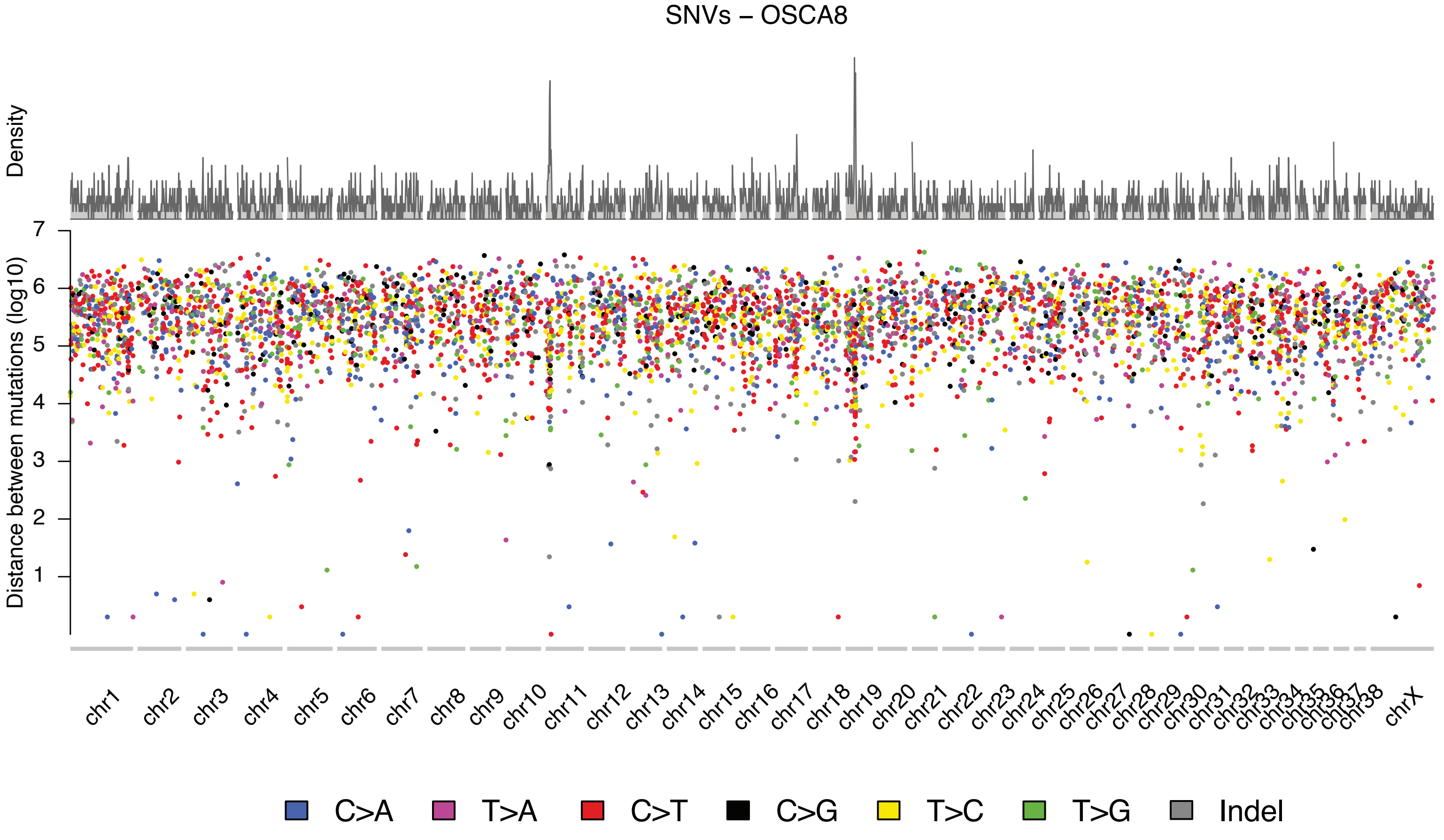
SNVs – OSCA8
Density
Distance between mutations (log10)
1
2
3
4
5
6
7
chr1 chr2 chr3 chr4 chr5 chr6 chr7 chr8 chr9 chr10 chr11 chr12 chr13 chr14 chr15 chr16 chr17 chr18 chr19 chr20 chr21 chr22 chr23 chr24 chr25 chr26 chr27 chr28 chr29 chr30 chr31 chr32 chr33 chr34 chr35 chr36 chr37 chr38 chrX
C>A
T>A
C>T
C>G
T>C
T>G
Indel
