# Supplementary material for: The genomic landscape of canine osteosarcoma cell lines reveals conserved structural complexity and pathway alterations
Source: PLoS One. 2022 Sep 13;17(9):e0274383. doi: 10.1371/journal.pone.0274383 (PMC9469990; doi:10.1371/journal.pone.0274383)
Supplement: S4 Fig — Mutation signatures and signature composition of each cell line. (PDF) [file pone.0274383.s004.pdf]

Supplemental Figure 4

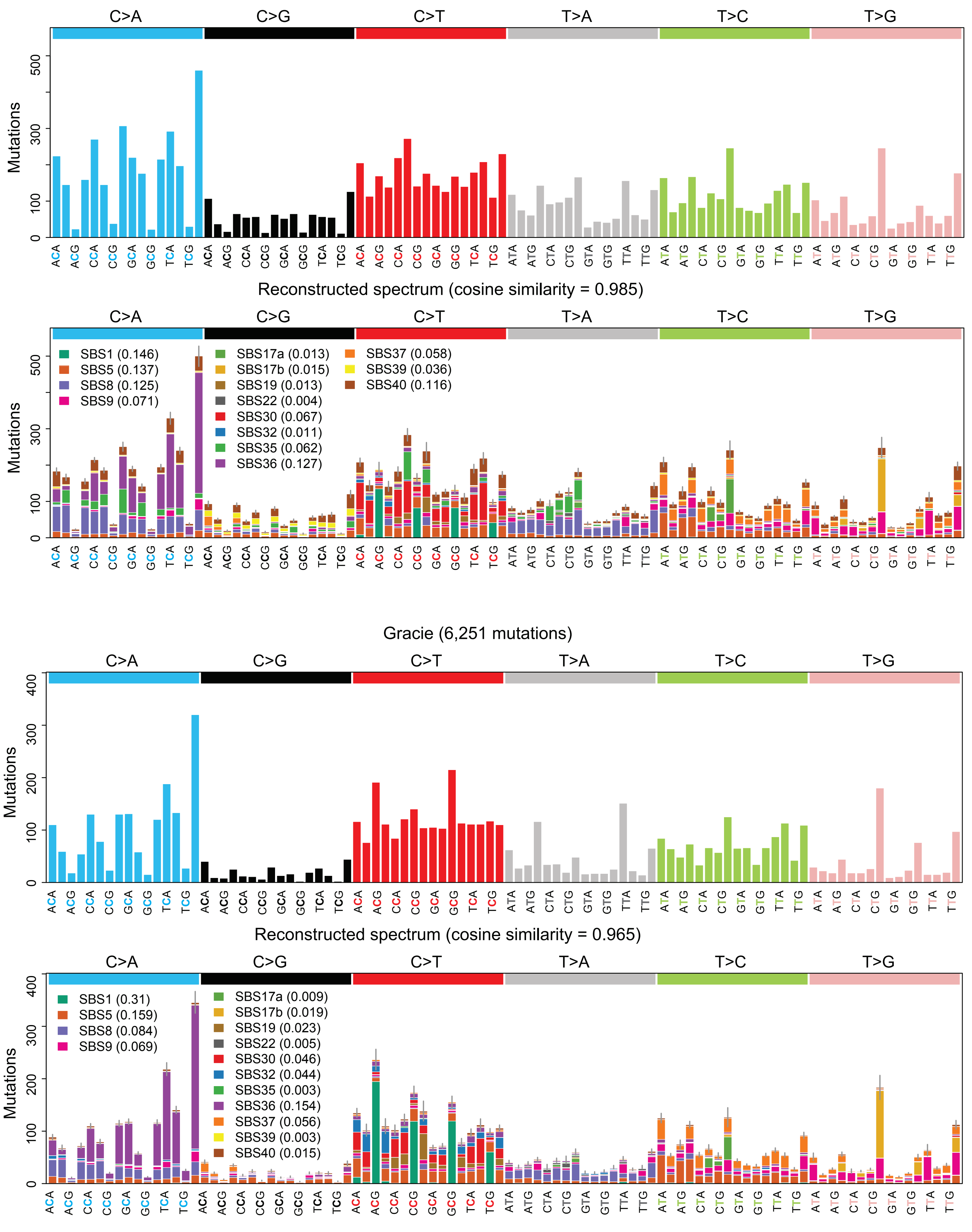

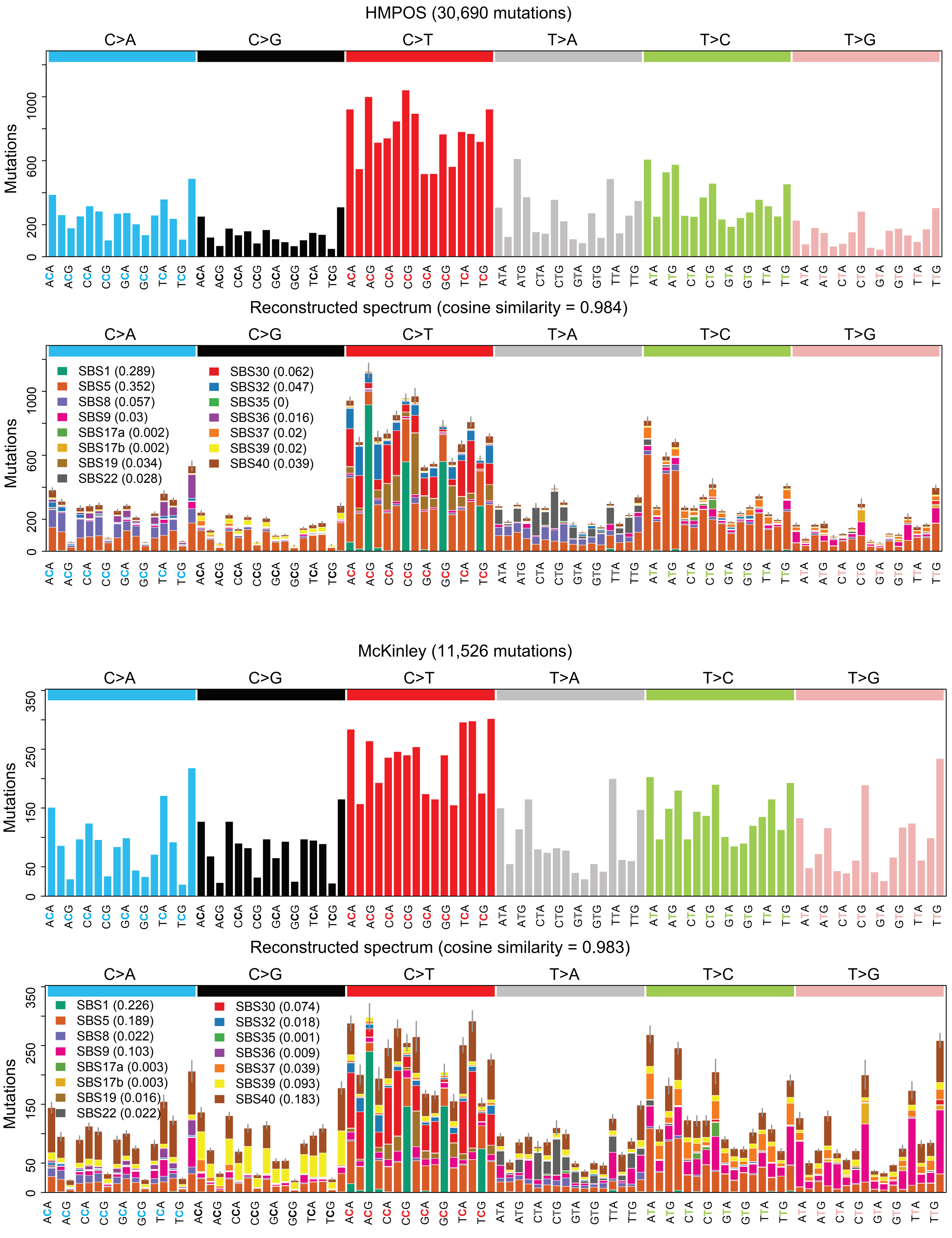
HMPOS (30,690 mutations)
C>A
C>G
C>T
T>A
T>C
T>G
Mutations
Reconstructed spectrum (cosine similarity = 0.984)
SBS1 (0.289)
SBS5 (0.352)
SBS8 (0.057)
SBS9 (0.03)
SBS17a (0.002)
SBS17b (0.002)
SBS19 (0.034)
SBS22 (0.028)
SBS30 (0.062)
SBS32 (0.047)
SBS35 (0)
SBS36 (0.016)
SBS37 (0.02)
SBS39 (0.02)
SBS40 (0.039)
McKinley (11,526 mutations)
Reconstructed spectrum (cosine similarity = 0.983)
SBS1 (0.226)
SBS5 (0.189)
SBS8 (0.022)
SBS9 (0.103)
SBS17a (0.003)
SBS17b (0.003)
SBS19 (0.016)
SBS22 (0.022)
SBS30 (0.074)
SBS32 (0.018)
SBS35 (0.001)
SBS36 (0.009)
SBS37 (0.039)
SBS39 (0.093)
SBS40 (0.183)

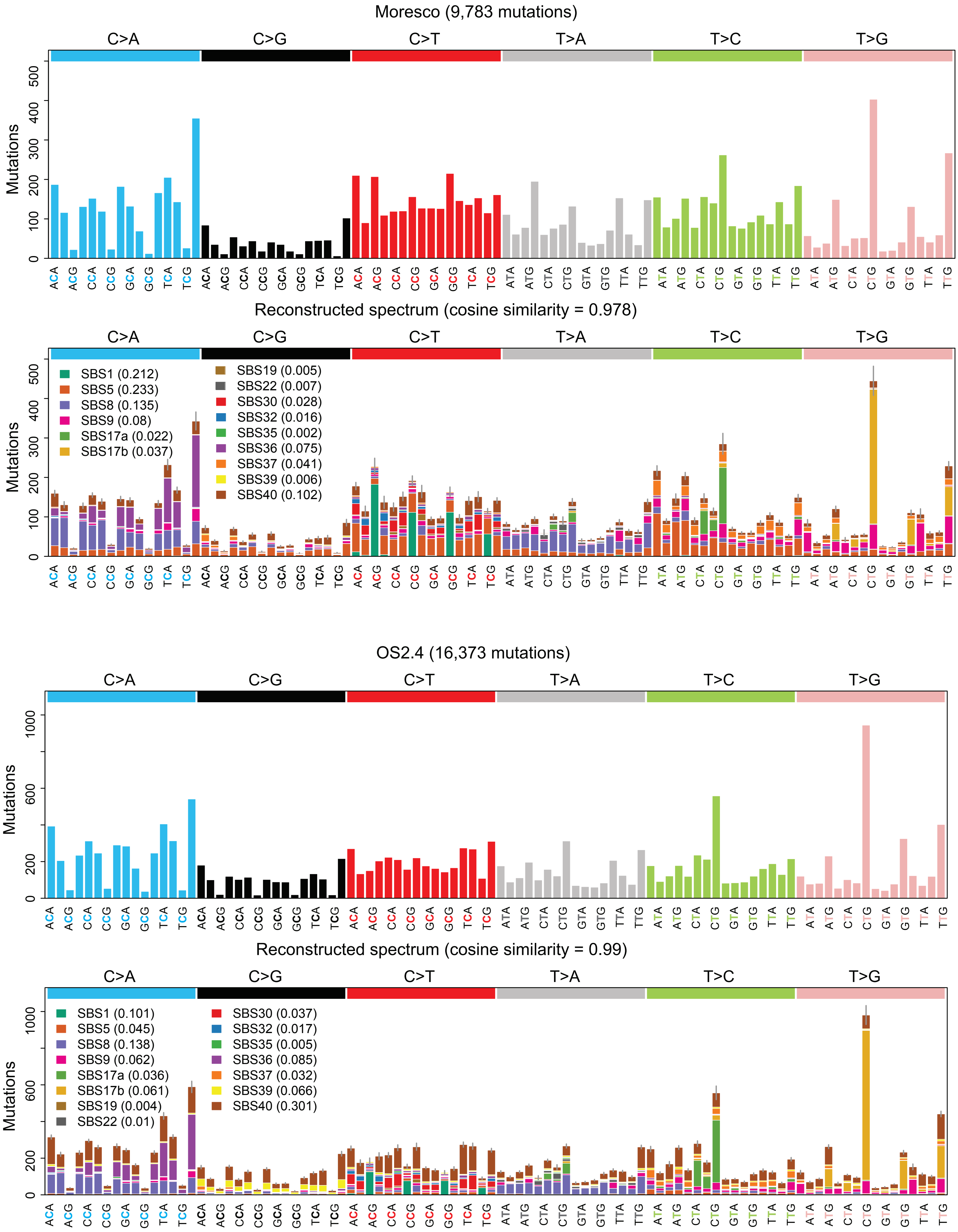

Moresco (9,783 mutations)
C>A
C>G
C>T
T>A
T>C
T>G
Mutations
Reconstructed spectrum (cosine similarity = 0.978)
SBS1 (0.212)
SBS5 (0.233)
SBS8 (0.135)
SBS9 (0.08)
SBS17a (0.022)
SBS17b (0.037)
SBS19 (0.005)
SBS22 (0.007)
SBS30 (0.028)
SBS32 (0.016)
SBS35 (0.002)
SBS36 (0.075)
SBS37 (0.041)
SBS39 (0.006)
SBS40 (0.102)
OS2.4 (16,373 mutations)
Reconstructed spectrum (cosine similarity = 0.99)
SBS1 (0.101)
SBS5 (0.045)
SBS8 (0.138)
SBS9 (0.062)
SBS17a (0.036)
SBS17b (0.061)
SBS19 (0.004)
SBS22 (0.01)
SBS30 (0.037)
SBS32 (0.017)
SBS35 (0.005)
SBS36 (0.085)
SBS37 (0.032)
SBS39 (0.066)
SBS40 (0.301)

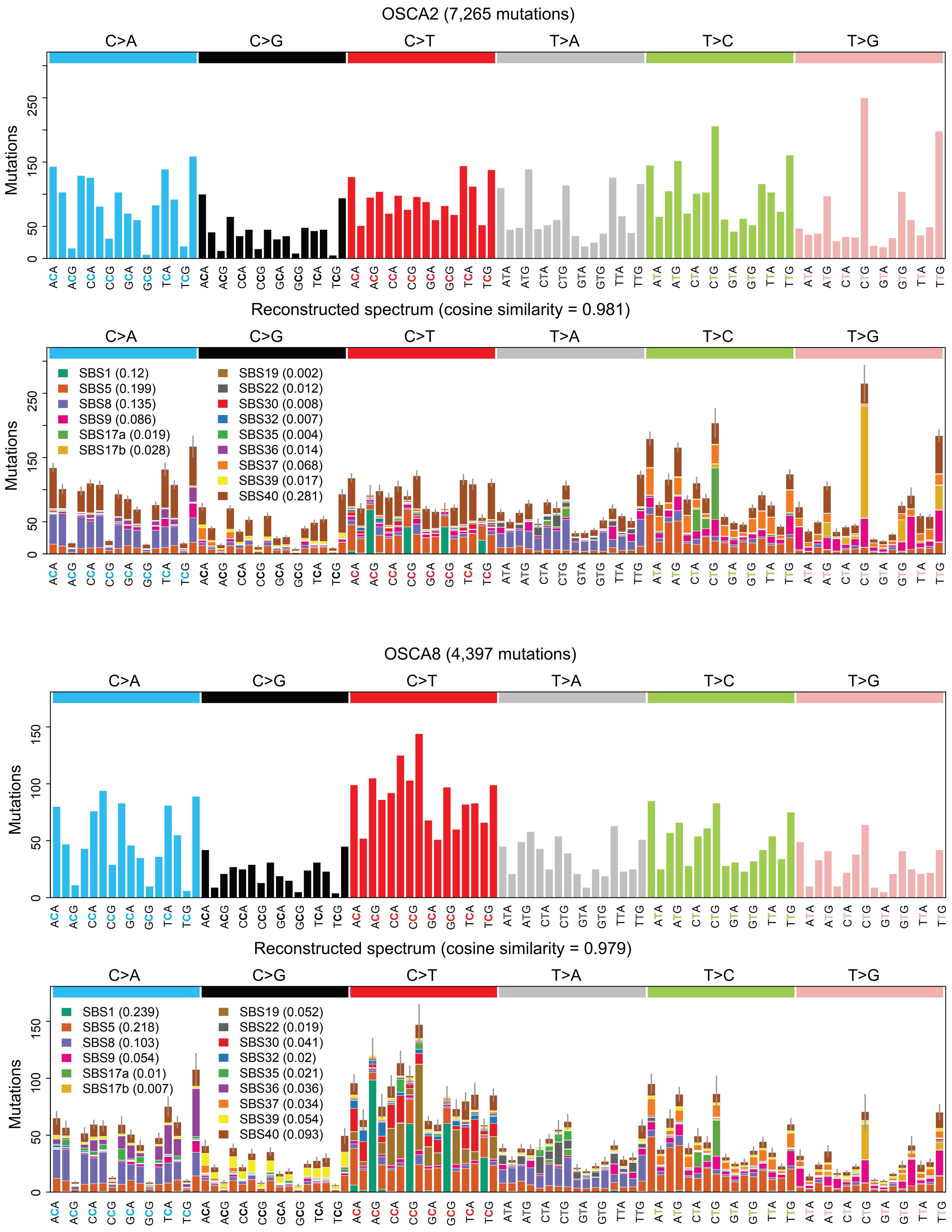

OSCA2 (7,265 mutations)
C>A
C>G
C>T
T>A
T>C
T>G
Mutations
Reconstructed spectrum (cosine similarity = 0.981)
SBS1 (0.12)
SBS5 (0.199)
SBS8 (0.135)
SBS9 (0.086)
SBS17a (0.019)
SBS17b (0.028)
SBS19 (0.002)
SBS22 (0.012)
SBS30 (0.008)
SBS32 (0.007)
SBS35 (0.004)
SBS36 (0.014)
SBS37 (0.068)
SBS39 (0.017)
SBS40 (0.281)
OSCA8 (4,397 mutations)
Reconstructed spectrum (cosine similarity = 0.979)
SBS1 (0.239)
SBS5 (0.218)
SBS8 (0.103)
SBS9 (0.054)
SBS17a (0.01)
SBS17b (0.007)
SBS19 (0.052)
SBS22 (0.019)
SBS30 (0.041)
SBS32 (0.02)
SBS35 (0.021)
SBS36 (0.036)
SBS37 (0.034)
SBS39 (0.054)
SBS40 (0.093)
