# Supplementary material for: The genomic landscape of canine osteosarcoma cell lines reveals conserved structural complexity and pathway alterations
Source: PLoS One. 2022 Sep 13;17(9):e0274383. doi: 10.1371/journal.pone.0274383 (PMC9469990; doi:10.1371/journal.pone.0274383)

Supplemental Figure 5

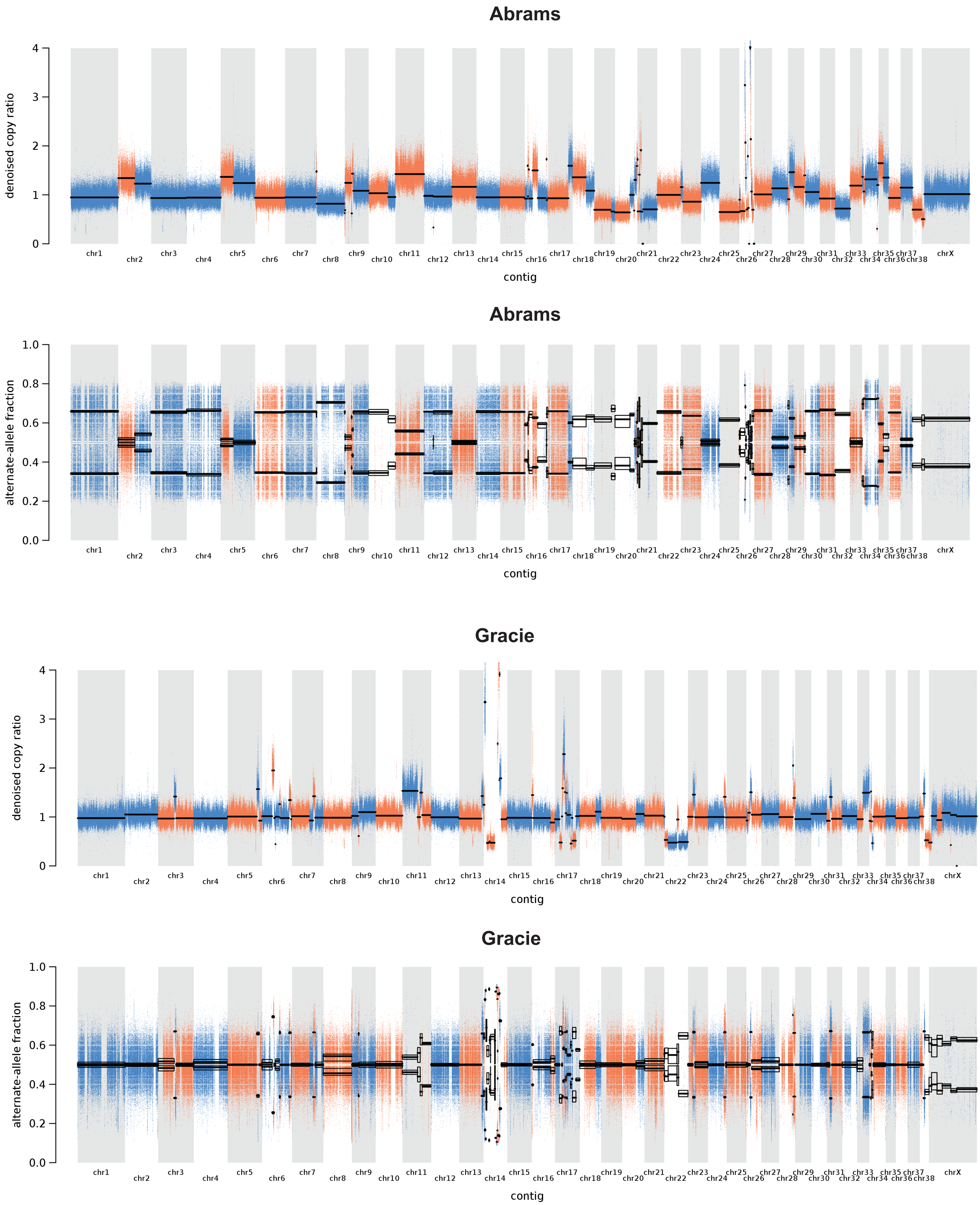

HMPOS

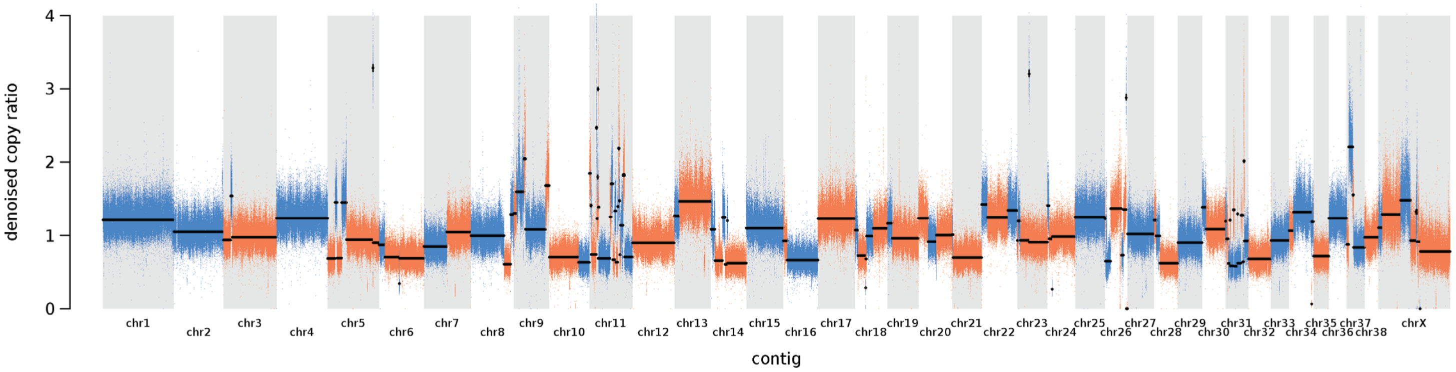

HMPOS

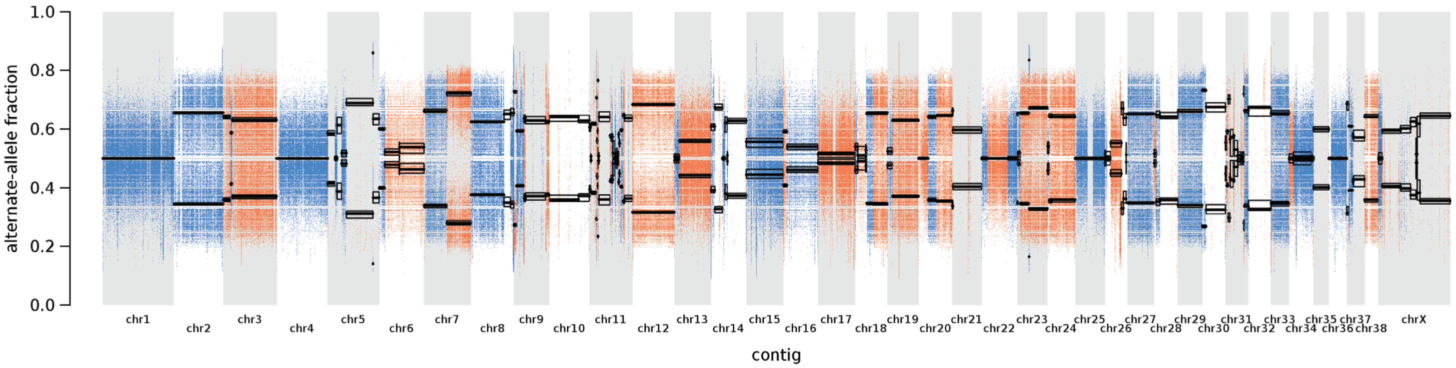

Moresco

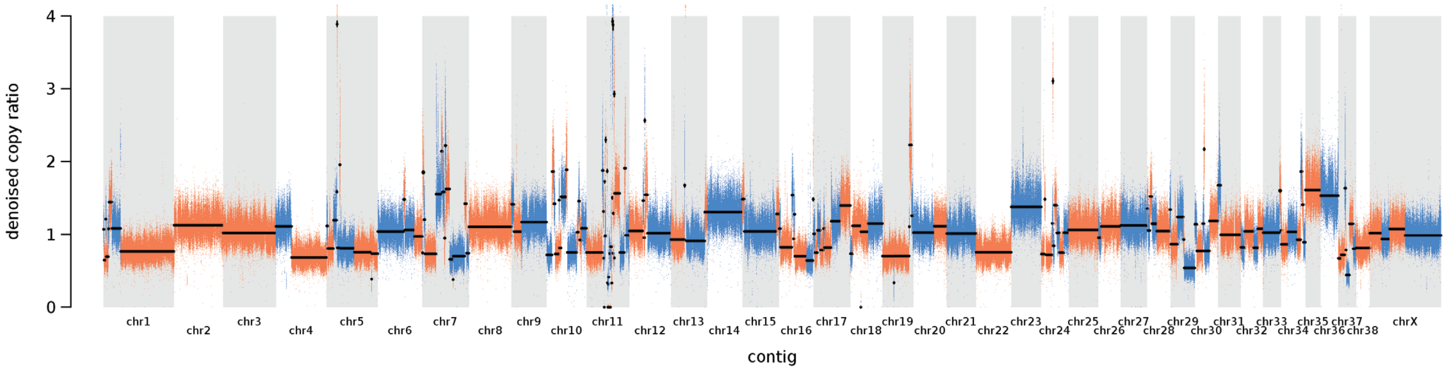

Moresco

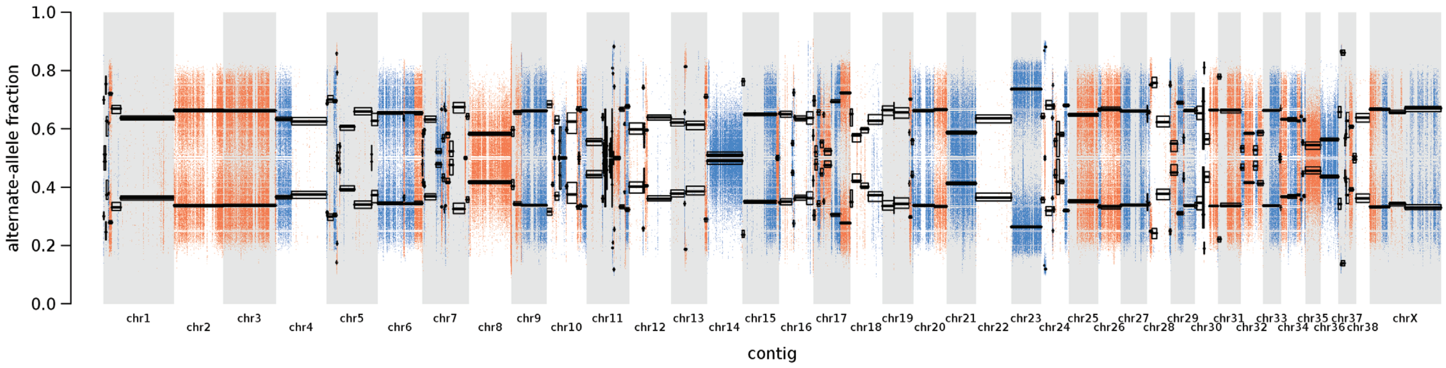

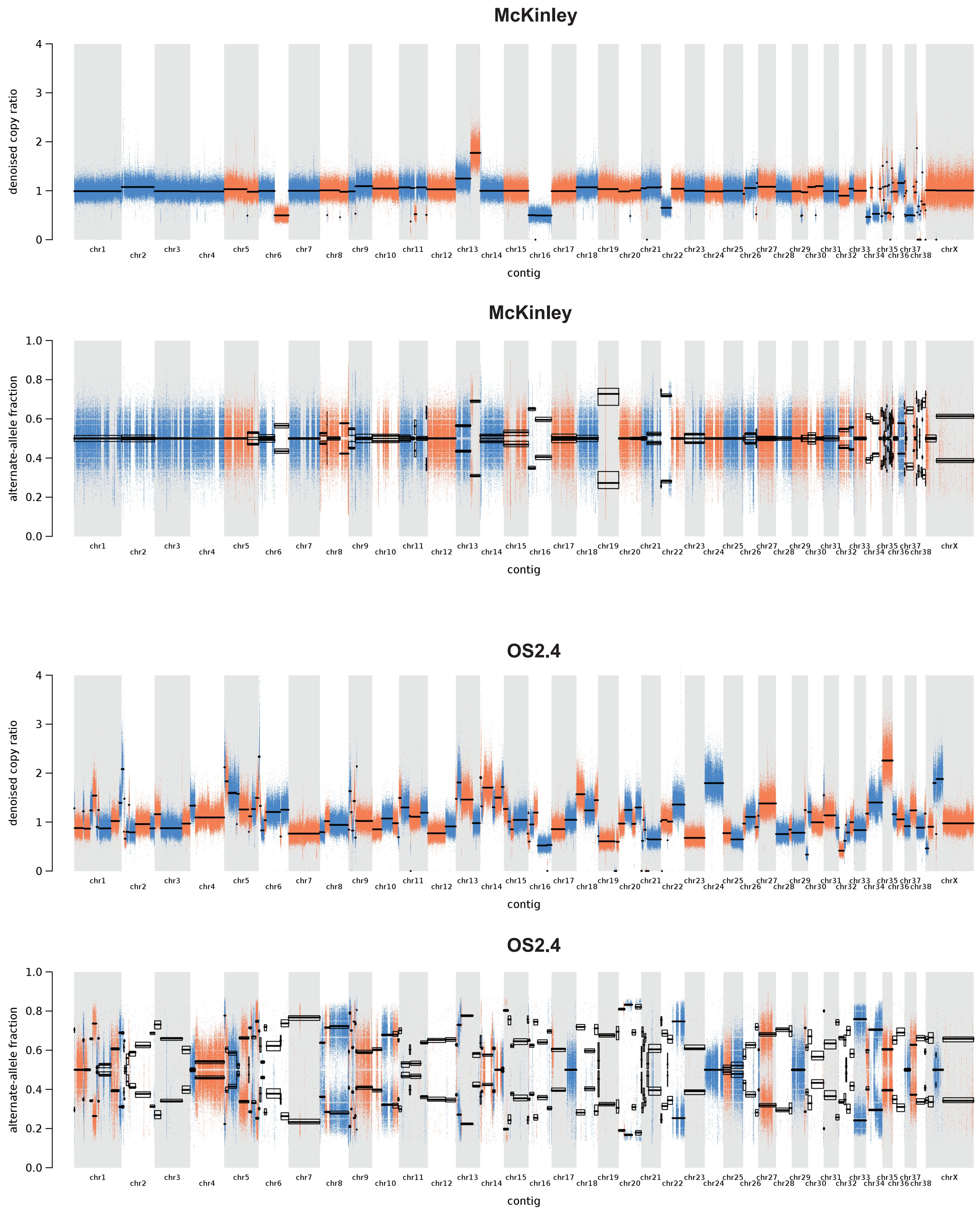
McKinley
denoised copy ratio
0
1
2
3
4
contig
McKinley
alternate-allele fraction
0.0
0.2
0.4
0.6
0.8
1.0
contig
OS2.4
denoised copy ratio
0
1
2
3
4
contig
OS2.4
alternate-allele fraction
0.0
0.2
0.4
0.6
0.8
1.0
contig
chr1 chr2 chr3 chr4 chr5 chr6 chr7 chr8 chr9 chr10 chr11 chr12 chr13 chr14 chr15 chr16 chr17 chr18 chr19 chr20 chr21 chr22 chr23 chr24 chr25 chr26 chr27 chr28 chr29 chr30 chr31 chr32 chr33 chr34 chr35 chr36 chr37 chr38 chrX

OSCA-2

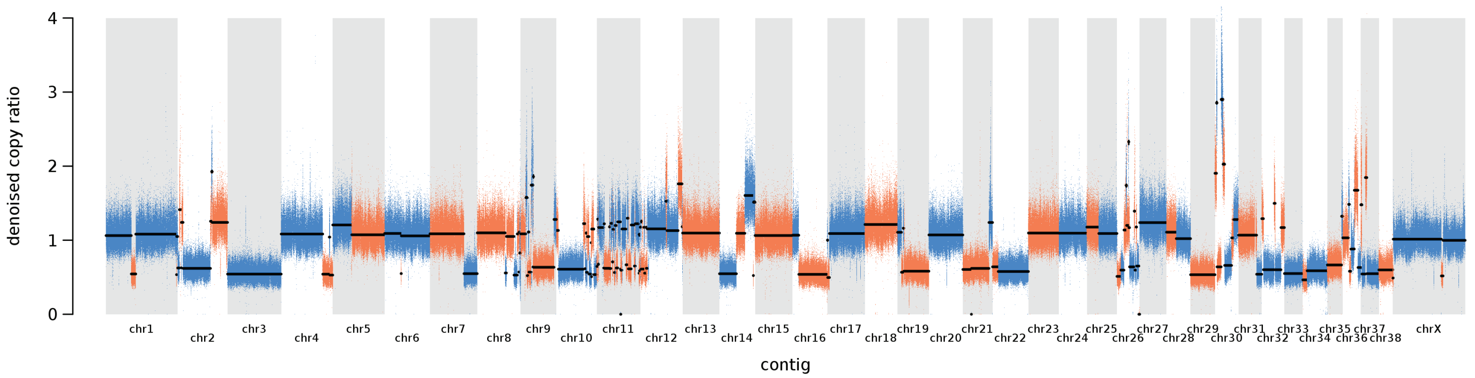

OSCA-2

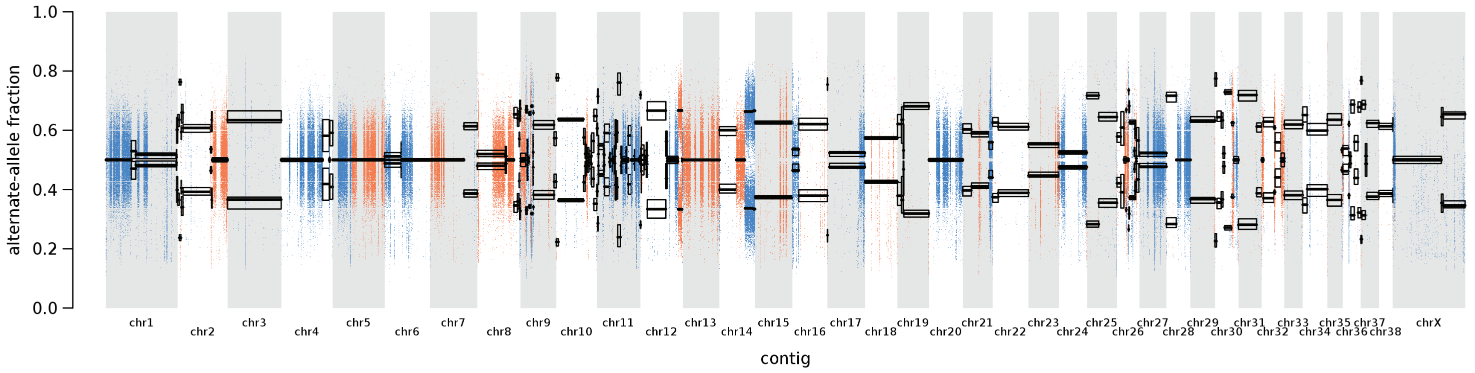

OSCA-8

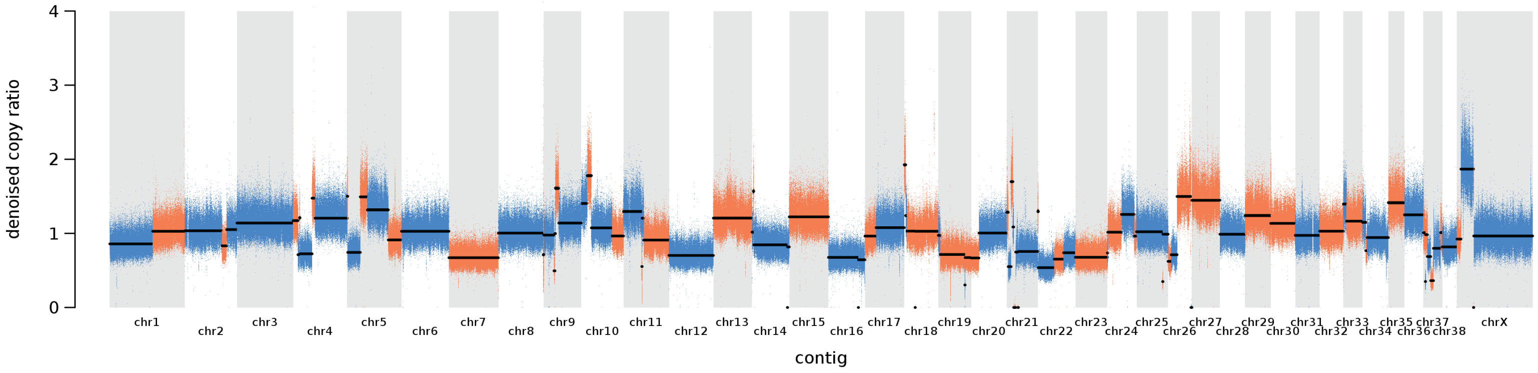

OSCA-8

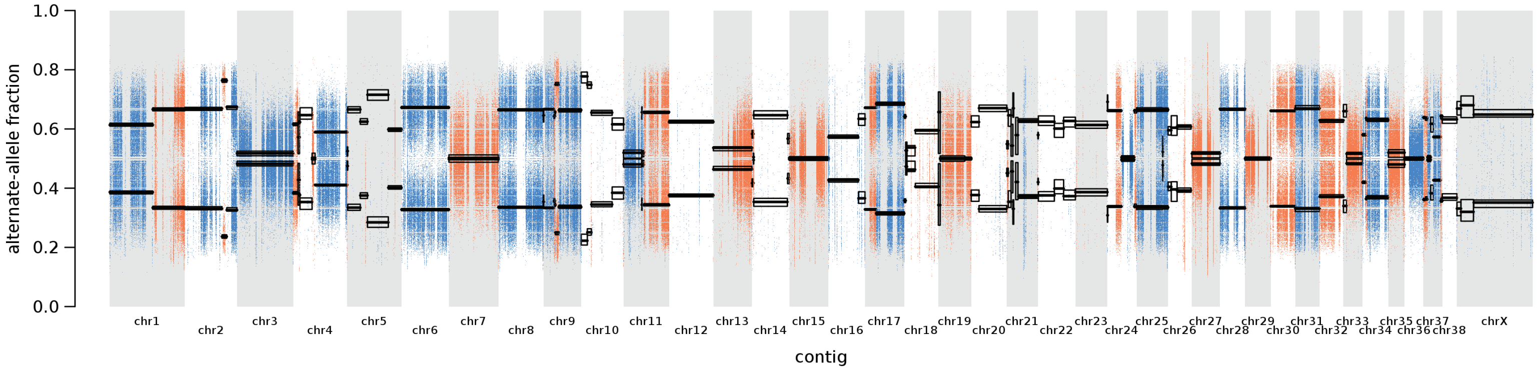

Supplement: S5 Fig — Denoised copy number segmentation plots and alternate-allele fraction ratios for each cell line. Copy ratio segments are highlighted alternating between blue and orange, while the denoised median is represented by the black lines. (PDF) [file pone.0274383.s005.pdf]
